# Tissue-Restricted Expression of Nrf2 and Its Target Genes in Zebrafish with Gene-Specific Variations in the Induction Profiles

**DOI:** 10.1371/journal.pone.0026884

**Published:** 2011-10-25

**Authors:** Hitomi Nakajima, Yaeko Nakajima-Takagi, Tadayuki Tsujita, Shin-Ichi Akiyama, Takeshi Wakasa, Katsuki Mukaigasa, Hiroshi Kaneko, Yutaka Tamaru, Masayuki Yamamoto, Makoto Kobayashi

**Affiliations:** 1 Institute of Basic Medical Sciences, Graduate School of Comprehensive Human Sciences, University of Tsukuba, Tsukuba, Japan; 2 Environmental Response Project, Japan Science and Technology Agency, University of Tsukuba, Tsukuba, Japan; 3 Graduate School of Bioresources, Mie University, Tsu, Japan; 4 Department of Medical Biochemistry, Tohoku University Graduate School of Medicine, Sendai, Japan; University College Dublin, Ireland

## Abstract

The Keap1-Nrf2 system serves as a defense mechanism against oxidative stress and electrophilic toxicants by inducing more than one hundred cytoprotective proteins, including antioxidants and phase 2 detoxifying enzymes. Since induction profiles of Nrf2 target genes have been studied exclusively in cultured cells, and not in animal models, their tissue-specificity has not been well characterized. In this paper, we examined and compared the tissue-specific expression of several Nrf2 target genes in zebrafish larvae by whole-mount *in situ* hybridization (WISH). Seven zebrafish genes (*gstp1*, *mgst3b*, *prdx1*, *frrs1c*, *fthl*, *gclc* and *hmox1a*) suitable for WISH analysis were selected from candidates for Nrf2 targets identified by microarray analysis. Tissue-restricted induction was observed in the nose, gill, and/or liver for all seven genes in response to Nrf2-activating compounds, diethylmaleate (DEM) and sulforaphane. The Nrf2 gene itself was dominantly expressed in these three tissues, implying that tissue-restricted induction of Nrf2 target genes is defined by tissue-specific expression of Nrf2. Interestingly, the induction of *frrs1c* and *gclc* in liver and nose, respectively, was quite low and that of *hmox1a* was restricted in the liver. These results indicate the existence of gene-specific variations in the tissue specificity, which can be controlled by factors other than Nrf2.

## Introduction

Nrf2 is a transcription factor that binds to the antioxidant response element (ARE) and transactivates cytoprotective genes [1], [2]. In basal conditions, Nrf2 is degraded via the Keap1-dependent proteasome pathway, while it is stabilized after cells are exposed to electrophilic or oxidative stress, which transactivates its target genes. Many studies have identified the Keap1-Nrf2 system to have multiple sensor sites to a variety of stresses [3], [4] and more than one hundred target genes [5], [6]. Conservation of the Keap1-Nrf2 system has been demonstrated in vertebrates including zebrafish [7], [8], which is a well-established research model.

Through previous studies, we noticed that the expression of *gstp1*, a major target gene for zebrafish Nrf2, is not ubiquitous as expected, but is rather restricted in the nose, gill, and liver [8]–[13]. It is unclear whether this tissue-restricted induction is specific for *gstp1* or a common feature for Nrf2 target genes, since *gstp1* was the only gene available in our study that was suitable for WISH analysis. In addition, most studies related to Nrf2 target genes have been performed on cultured cells or a specific murine tissue, and therefore the tissue specificity of Nrf2 target genes has not yet been well characterized, even in other animals. In this paper, we identified seven zebrafish genes appropriate for WISH using microarray analysis and examined their tissue-specific expression in zebrafish larvae. Induced expression of all these genes was restricted to the nose, gill, and/or liver with gene-specific variations in tissue specificity. These results indicate tissue-restricted induction to therefore be a common feature of Nrf2 target genes, which can be a critical issue for both the pharmacological and clinical applications of Nrf2-activating compounds.

## Materials and Methods

### RT-PCR and WISH analyses

Zebrafish embryos and larvae were obtained by natural mating. All experiments were carried out using a wild-type AB strain. For induction studies, embryos or larvae were placed in culture dishes containing 100 µM DEM (Wako, Osaka, Japan) or 40 µM sulforaphane (LKT laboratories, St. Paul, MN). RT-PCR analysis was performed as described previously [8] using PCR primers listed in Table S1. WISH analysis was carried out as described previously [14] with slight modifications in the fixation step. Briefly, the larvae were fixed with 4% paraformaldehyde (PFA) in PBS overnight at 4°C, and washed twice in PBS, once in 50% methanol, and twice in 100% methanol, and cooled to –20°C for at least 3 hours. Fixed larvae were then brought back to room temperature (RT), washed twice in PBT (0.1% Tween 20/PBS) for 5 minutes, and immersed for 2 hours in 9% hydrogen peroxide in PBT. After immersion, the larvae were washed twice in PBTw (0.2% bovine serum albumin in PBT), treated for 20 minutes with 50 µg/ml proteinase K (Sigma-Aldrich, St. Louis, MO), and fixed with 4% PFA/PBS for 20 minutes at RT.

### Microarray analysis

A microarray analysis was performed using custom-made 16 K MZH chips. The MZH chips contained in a total of 16399 probes of a 65 oligonucleotides in length purchased from Sigma-Aldrich. The collected zebrafish embryos were quickly homogenized with 1 ml of QIAzol reagent (Qiagen, Hilden, Germany), and subsequently stored at -80°C. Total RNA was extracted according to the manufacturer’s instructions. Isolated total RNA was then further purified using the RNAeasy mini kit (Qiagen). Amino-allyl-modified amplified RNA was synthesized in one amplification round from 1 µg of purified total RNA using the amino-allyl RNA amplification kit (Sigma-Aldrich). Subsequently, 5 µg of amino-allyl-modified amplified RNA was used for coupling of monoreactive Cy3 and Cy5dyes (GE Healthcare, Little Chalfont, UK) and column purified. Dual-color hybridization of the MZH chips was performed according to the manufacturer’s instructions for the AceGene DNA microarray (Hitachi Solutions, Tokyo, Japan). Each experiment was repeated in triplicate. After hybridization, MZH chips were scanned using the Affymetrix 428 array scanner (Affymetrix, Santa Clara, CA). The microarray data were processed from raw data image files with Affymetrix Jaguar (Affymetrix) and normalized. The processed data were subsequently imported into Excel (Microsoft, Redmond, WA) to compare expression profiles of DEM-treated samples to control samples. The cut-offs for significance regarding the ratios of DEM-treated samples vs control samples were set at a 2-fold change.

We have deposited the raw data at GEO under accession numbers (GSM799460, GSM799461, GSM799462, GSM799463), and we can confirm all details are MIAME compliant.

### Plasmid construction

cDNA clones were prepared of the following transcripts by RT-PCR using total RNA from 5 days post-fertilization (dpf) zebrafish larvae: *gstal*, *zgc:158387* (*mgst3b*), *sepw2b*, *bcat1*, *zgc:110343* (*prdx1*), *zgc:163022* (*frrs1c*), *zgc:92066* (*fthl*), *gclc*, *gclm*, and *hmox1* (*hmox1a*). Specific primers were designed based on cDNA information (http://zfin.org), and cDNA products were subcloned into the pBluescript II SK vector (Table S2). Plasmids pCS2nrf2, pCS2FLmKeap1, and pKSgstp1N have been described previously [8], [9], [12].

### Knockdown and overexpression analyses of Nrf2

Synthetic capped *nrf2* RNA was made with an SP6 mMESSAGE mMACHINE in vitro transcription kit (Ambion, Austin, TX) using pCS2*nrf2*. The Nrf2-morpholino oligonucleotide (*nrf2*MO) has been described previously [9]. mRNA or morpholino oligonucleotides were injected into yolk of the zebrafish at the one-cell stage using an IM300 microinjector (Narishige, Tokyo, Japan).

### Sectioning of zebrafish larvae

After carrying out WISH analysis, larvae were fixed with 4% PFA/PBS, embedded in 1.5% SeaPlaque GTG agarose (Takara Bio, Osaka, Japan), and dehydrated through graded ethanol series (30%, 50%, 90% and twice 100%) in PBS for 15 minutes each. Glycol methacrylate (Technovit 8100; Heraeus Kulzer, Wehrheim, Germany), with low-temperature polymerization, was used according to the manufacturer's instructions. After embedding, 6-µm serial sections were made from whole bodies of zebrafish larvae with an RM 2045 microtome (Leica, Wetzler, Germany).

## Results

### Identification of Nrf2 target genes in zebrafish

In order to investigate tissue-specific expression of Nrf2 target genes, we searched for zebrafish Nrf2 targets other than *gstp1* that were able to be used for WISH analysis. Microarray analysis was carried out using cDNA prepared from 4-dpf larvae treated with or without 100 µM DEM for 12 hours. In total, 16,000 zebrafish cDNAs were screened and 42 genes were identified that showed more than a 2-fold induction compared with DEM-treated larvae and untreated larvae (Table S3). The reliability of this screen was demonstrated by the fact that *gstp1* produced a top ranking score in this analysis. In microarray analysis, *gclm* and *nqo1* were induced by DEM at levels of only 1.97- and 1.93-fold, respectively. Since they have generally been used as Nrf2 target genes in mammalian cells, we selected them together with *gclc* and *hmox1*, for further analysis, in addition to 42 identified genes (Table S4).

**Figure 1:**
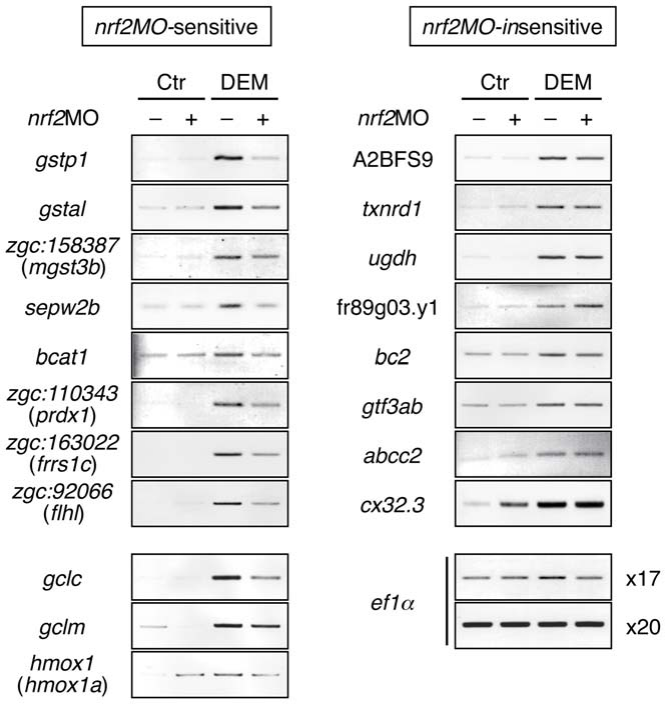
Screening of Nrf2 target genes in zebrafish. DEM-induced expression of candidate genes for Nrf2 target analyzed by RT-PCR. Embryos were injected with or without *nrf2*MO and treated with or without 100 µM DEM for six hours at 2 hours post-fertilization (hpf) using total RNA from the whole bodies.

We next carried out RT-PCR analysis to confirm the results of microarray analysis. RT-PCR analysis was performed using RNA isolated from 4-dpf larvae treated with DEM and isolated RNA (Figure 1 and Table S3). Thirty-three genes were analyzed out of 46 selected genes, and 19 genes were identified to be induced by DEM. To clarify whether these inductions were directed by Nrf2, we carried out RT-PCR analysis using Nrf2-knockdown larvae using *nrf2*-specific morpholino oligonucleotide. As a result, the induction of eleven genes was clearly demonstrated to be Nrf2-dependent (Figure 1).

We constructed phylogenetic trees of the 11 identified genes, with the exception of *gstp1*, using the zebrafish genome and cDNA information; we also renamed some genes (Figures S1–S10). Finally, the genes were identified as *gstal* (glutathione *S*-transferase alpha like), *mgst3b* (microsomal glutathione *S*-transferase 3b), *sepw2b* (selenoprotein W2b), *bcat1* (branched chain aminotransferase 1), *prdx1* (peroxiredoxin 1), *frrs1c* (ferric-chelate reductase 1c), *fthl* (ferritin heavy chain like), *gclc* (glutamate-cysteine ligase catalytic subunit), *gclm* (glutamate-cysteine ligase modifier subunit) and *hmox1a* (heme oxygenase 1a). Gsta, Prdx1, Fth, Gclc, Gclm and Hmox1 have been identified as typical Nrf2 target genes in mammalian cells [15]–[19]. Mgst3 has also been suggested to be an Nrf2 target from several microarray studies [20]–[22]. These results indicated that target genes of Nrf2 are conserved among vertebrates. More interestingly, SepW2, Bcat1, and Frrs1 have never been indentified as Nrf2 targets, including from microarray analyses. Among these three, two are redox-regulating proteins which are possible candidates for Nrf2 targets: SepW2 is a member of the selenoprotein family, which has been shown to possess anti-oxidative stress activity [23], and Frrs1 is an iron-metabolizing enzyme that reduces ferric ion [24]. Bcat1 is a metabolizing enzyme for branched-chain amino acids and plays important roles in ammonia metabolism [25].

**Figure 2:**
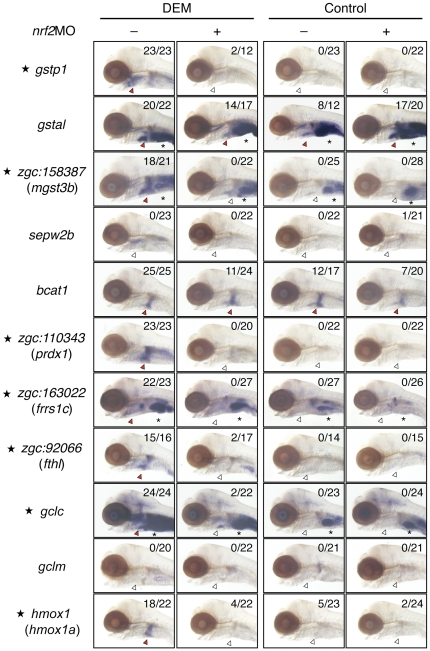
WISH analysis of Nrf2 target genes. Expression of eleven Nrf2 target genes was analyzed by WISH. Embryos were injected with or without *nrf2*MO and treated with or without 100 µM DEM for six hours (three hours only for *hmox1a*) at 5 dpf. Lateral views. Numbers indicate the induction positive embryos/tested embryos. Red and white arrowheads indicate positive and negative expression, respectively. Asterisks denote basal expression in the intestine.

A WISH analysis was carried out using eleven genes as probes (Figure 2). Among them, seven genes (*gstp1*, *mgst3b*, *prdx1*, *frrs1c*, *fthl*, *gclc* and *hmox1a*) showed clear and strong induction in response to DEM, suggesting they will be useful for studying tissue specificity of Nrf2 target genes. The remaining four genes showed either weak induction (*sepw2b* and *gclm*) or strong constitutive expression (*gstal* and *bcat1*), thus indicating that they were unsuited for gene expression studies. We, therefore, used the seven genes showing a strong induction for further analyses.

### Tissue-restricted induction of Nrf2 target genes

**Figure 3:**
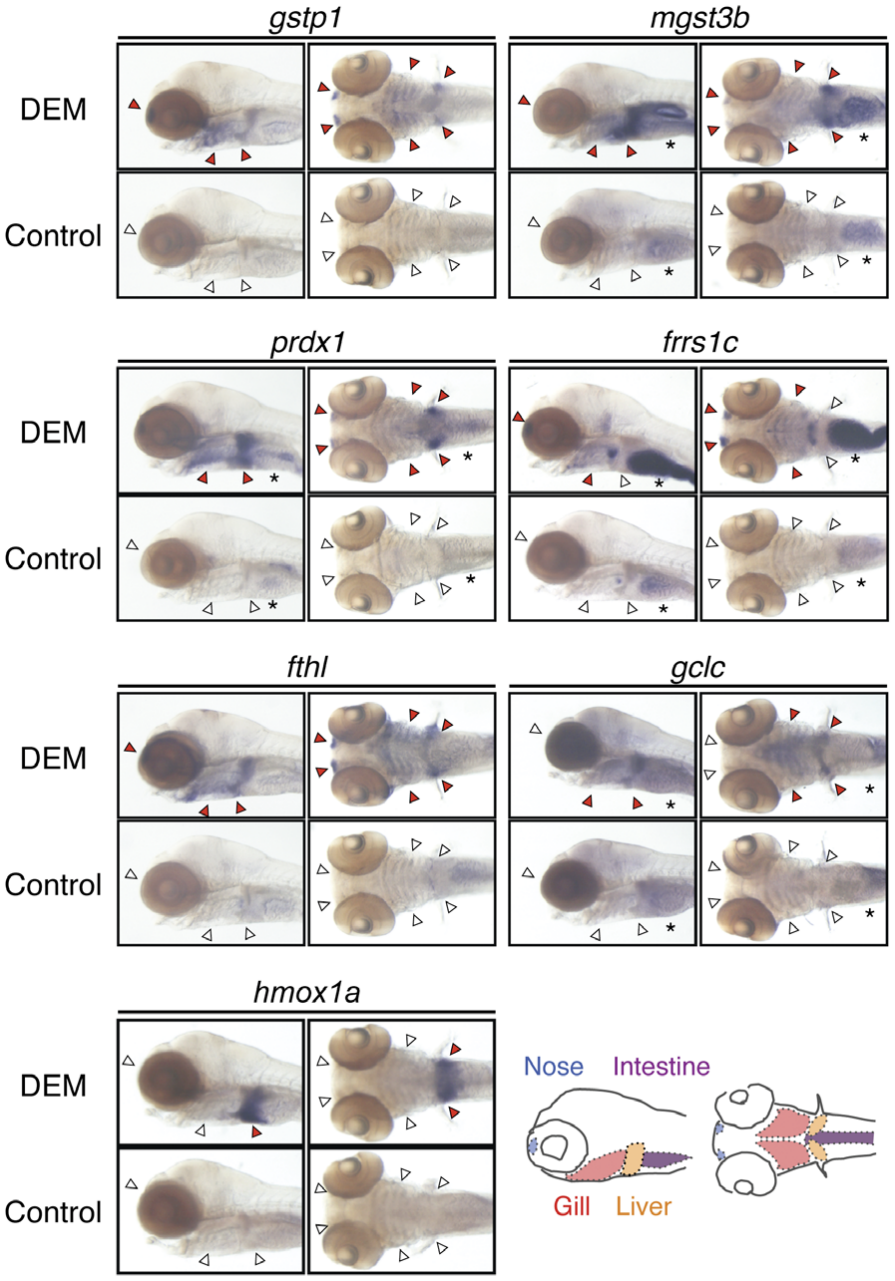
Tissue-restricted induction of Nrf2 target genes. 5-dpf larvae were treated with or without 100 µM DEM for six hours (three hours only for *hmox1a*) and expression of indicated genes was analyzed by WISH. Lateral and ventral views. Red and white arrowheads indicate positive and negative expression, respectively, of each gene in the nose, gill and liver. Asterisks denote basal expression in the intestine.

Tissue-restricted induction of seven Nrf2 target genes was examined by WISH (Figures 3 and S11), using 5-dpf larvae, since the induction was much clearer compared with that in 4-dpf larvae. As a result, the induction of *mgst3b*, *prdx1*, *frrs1c*, *fthl*, and *gclc* was observed in nose, gill, and liver, similar to *gstp1*, although the expression of *frrs1c* and *gclc* in the liver and nose, respectively, was relatively weak. Induction in the intestine was observed in the case of *mgst3b*, *prdx1*, *frrs1c* and *gclc*, but we did not take them into account since a considerable level of basal expression was detected. Interestingly, *hmox1a* was only induced in the liver, suggesting the existence of gene-specific differences in tissue specificity. Expression profiles of *hmox1a* and *frrs1c* in the liver were confirmed by section analysis in comparison with a liver-specific marker *fabp10* (Figure S11) [26]. These results indicate that tissue-restricted induction is a common characteristic among Nrf2 target genes, not only for *gstp1*.

**Figure 4:**
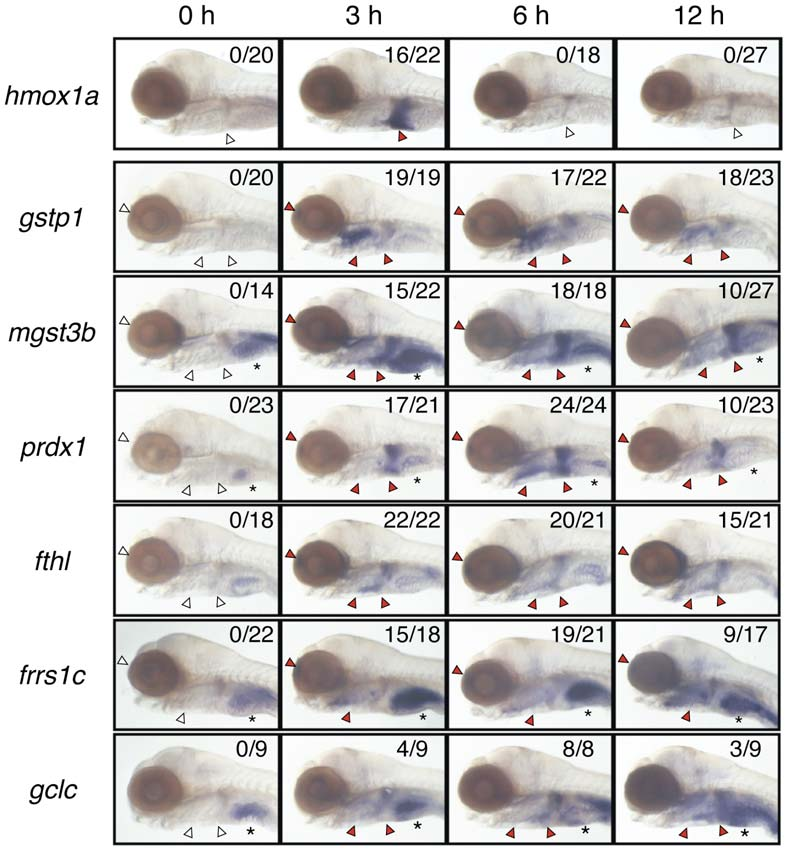
Induction time profiles of Nrf2 target genes. 5-dpf larvae were treated with 100 µM DEM for indicated hours and expression of seven genes was analyzed by WISH. Lateral views. Red and white arrowheads indicate positive and negative expression, respectively, of each gene in the nose, gill and liver. Asterisks denote the basal expression in the intestine.

The induction time profiles of seven genes were also analyzed in detail (Figure 4). All genes showed induction beginning three hours after DEM treatment. Interestingly, expression of *hmox1a* was rapidly reduced beginning six hours after DEM treatment to almost the same level as the non-induced condition, whereas expression levels of the remaining six genes were maintained until twelve hours after treatment. A negative feedback regulation may exist in the case of *hmox1a*.

To determine whether tissue specificity and induction time profiles of Nrf2 target genes vary according to differences of Nrf2-activating compounds, expression profiles of seven genes by sulforaphane were analyzed (Figures S12). Induction profiles of all seven genes were basically identical to those in the case of DEM, suggesting that tissue-restricted induction of Nrf2 target genes is intrinsic properties of each gene.

### Gene regulation by Nrf2

**Figure 5:**
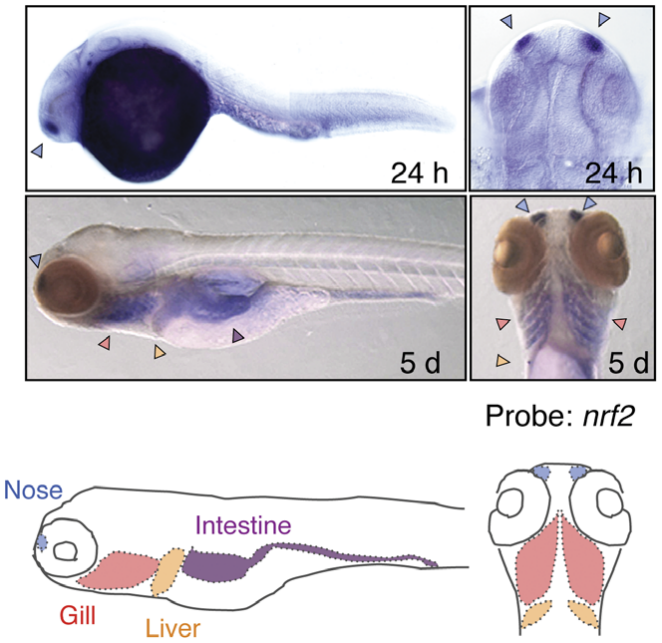
Tissue-specific expression of the Nrf2 gene. Expression of Nrf2 gene in 24-hpf embryos and 5-dpf larvae were analyzed by WISH. Lateral (upper left, lower left), dorsal (upper right), and ventral (lower right) views. Arrowheads in light blue, red and orange indicate expression in the nose, gill and liver.

Considering that all genes tested showed restricted-expression in the nose, gill, liver, and intestine, gene expression in these four tissues seemed to be a default state for Nrf2 target genes. This may suggest that Nrf2 or its activator dominantly exists in these tissues and directly transactivates the target genes. To test this hypothesis, we analyzed the expression of *nrf2* in 5-dpf larvae by WISH (Figure 5). As expected, *nrf2* is specifically expressed in the nose, gill, liver, and intestine, suggesting that tissue-restricted induction of Nrf2 target genes is based on the tissue-specific expression of *nrf2* mRNA.

**Figure 6:**
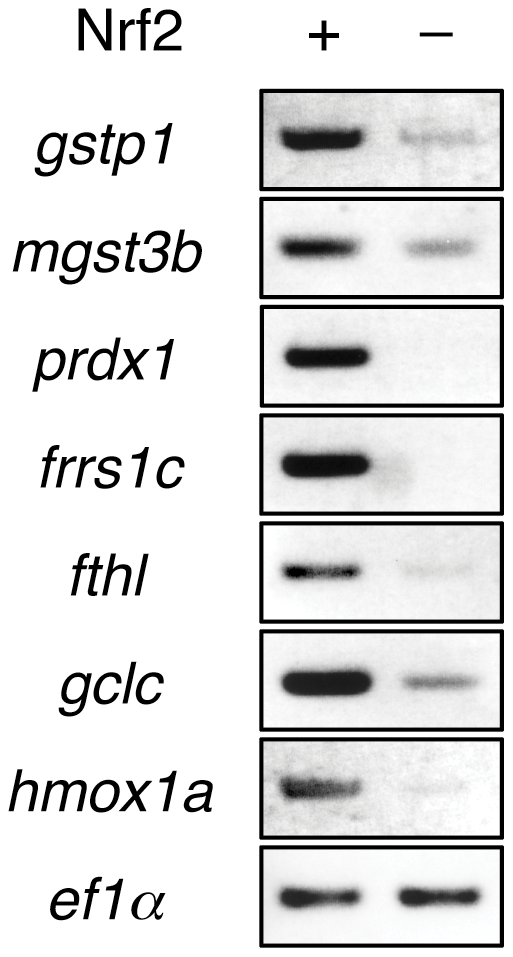
Target gene induction in zebrafish embryos by Nrf2 overexpression. RT-PCR analysis using total RNA from the whole bodies of 30 embryos and specific primers of indicated genes.

**Figure 7:**
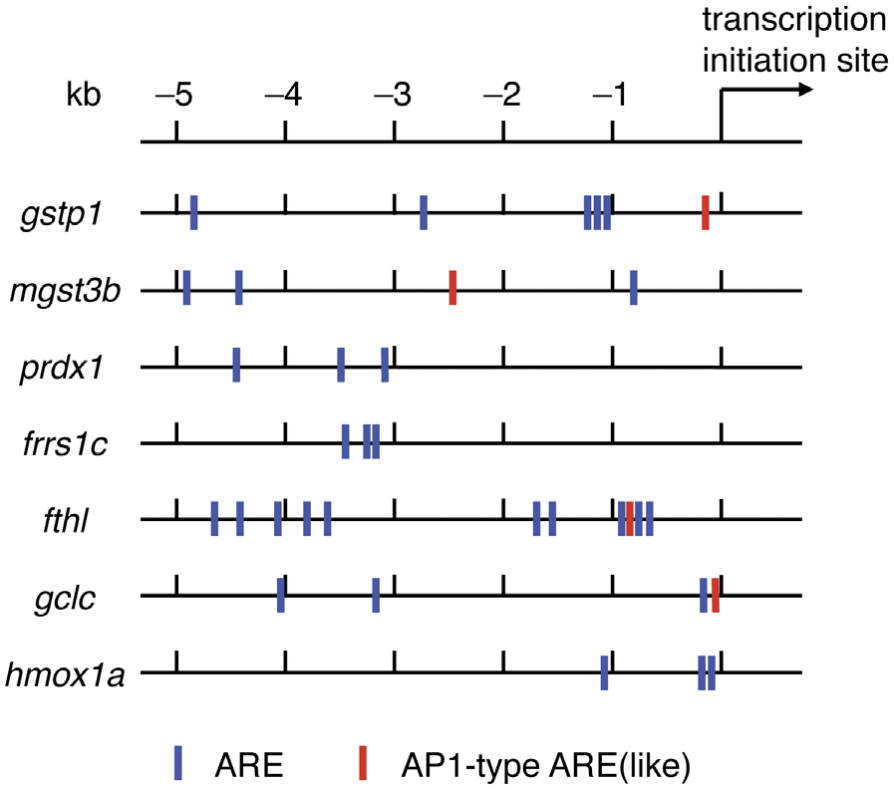
ARE sequences in upstream regions of Nrf2 target genes. ARE sequences (TGAG/CNNNGC) and AP1-type ARE (like) (TGAG/CTCAGN or TGAG/CTCANC) in the 5-kb region of deduced transcription initiation sites of indicated genes were searched using the zebrafish genome database.

We previously demonstrated that the expression of *gstp1* and *gclc* (γ*gcsh*) to be strongly induced when Nrf2 is overexpressed in zebrafish embryos [9]. We next investigated whether the other five Nrf2 targets can also be induced by Nrf2 overexpression. Nrf2 was overexpressed by injecting *nrf2* mRNA into one-cell-stage embryos, and the expression of Nrf2 target genes was analyzed by RT-PCR seven hours after injection (Figure 6). The results indicate that all seven genes were induced by Nrf2 overexpression. Using the zebrafish genome database (http://www.ensembl.org/Danio_rerio/Info/Index), we searched the ARE sequences in the 5-kb upstream regions of the deduced transcription initiation sites in these genes (Figure 7). All genes were found to have more than three ARE sites in the 5-kb regions, to which Nrf2 may bind and regulate. All of these results suggest that induction of these seven genes is directly regulated by Nrf2.

## Discussion

The zebrafish is a good system to observe the tissue-specific expression of many genes, including gene induction, as described in this study. Indeed, tissue-specific induction in systems other than Keap1-Nrf2, such as the heat-shock genes, has been reported from studies in zebrafish [27], [28]. These observations would be difficult to analyze using cultured cell lines, demonstrating a significant advantage for zebrafish system. Zebrafish is also good for drug toxicity screening and testing environmental toxicants [29], [30]. Since Nrf2 is activated by a variety of toxic compounds and oxidative stress, it would be worthwhile for these screens and tests to analyze tissue-restricted induction of Nrf2 target genes. The seven target genes of Nrf2 selected in this paper would be useful for such studies.

In our analysis, all tested genes showed tissue-restricted induction. Furthermore, we found gene-specific variation in tissue specificity, *e.g.*, a weak induction of *frrs1c* and *gclc* in the liver and nose, respectively, and no *hmox1a* induction in the nose and gill. The requirements of metabolizing enzymes encoded by each target gene may be different among tissues and the level of enzymes are controlled at a transcriptional level. An important finding in this study is that the expression level of Nrf2 itself is different among tissues, which will be the critical point to exert tissue-restricted induction of its target genes. We hypothesized that the gene expression profiles of the Nrf2 gene defines a default state of tissue specificity of target genes. In cases of *frrs1c*, *gclc* and *hmox1a*, some tissue- and gene-specific transcriptional repressors may be involved to exert their tissue-specific variations. Nrf2-dependent ARE gene regulation has been shown to be negatively regulated by other ARE-binding proteins such as Nrf1, Nrf3, Bach1, small Maf proteins, and c-Maf [31]–[35]. Since we have previously demonstrated that the target genes of Nrf-Maf proteins tend to differ somewhat among family proteins [36], [37], it is possible that these ARE-acting factors may bind in a gene-specific manner and interfere with DNA binding of Nrf2. Furthermore, ATF3, c-Myc and p53 have also been reported to repress Nrf2-dependent gene activation [38]–[40]. Some of these transcription factors may bind near the ARE in a gene- and tissue-specific manner and inhibit the transcription activity of Nrf2.

In contrast to Nrf2 regulation at a post translational step, molecular mechanisms of Nrf2 gene regulation have not been extensively studied. The exception is the upregulation of the Nrf2 gene by Nrf2 itself and aryl hydrocarbon receptor in response to their chemical activators [41], [42]. We previously reported that the embryonic expression of the Nrf2 gene is quite low both in the mouse and zebrafish and it becomes elevated near birth [12], [43]. The downregulation of Nrf2 was found in prostate cancer which may be related to the initiation of cellular transformation [44]. A low Nrf2 expression in the brain has been reported in humans, mice, and chickens [45]–[47], as was also the case in our present study. For the efficient medical applications of the Keap1-Nrf2 system for the treatment of neurodegenerative diseases [48], [49], Nrf2 should be considerably expressed in the brain. However, we found the expression of Nrf2 in the brain to be low. It will thus be valuable to find new methods and procedures to elevate the Nrf2 expression in both brain and prostate cancer cells.

## Supporting Information

Figure S1 **Phylogenetic tree of Gsta family proteins.** Amino acid sequences of full-length proteins were analyzed. The tree was constructed by the neighbor-joining method using the ClustalW program (http://clustalw.ddbj.nig.ac.jp/top-j.html). *c*, chicken; *h*, human; *m*, mouse; *r*, rat; *xt*, *Xenopus tropicalis*; *z*, zebrafish.
(TIF)

Figure S2 **Phylogenetic tree of Mgst3 family proteins.** Amino acid sequences of full-length proteins were analyzed. *ci*, *Ciona intestinalis*; *x*, *Xenopus laevis*.
(TIF)

Figure S3 **Phylogenetic tree of SepW family proteins.** Amino acid sequences of full-length proteins were analyzed. *d*, *Drosophila melanogaster*.
(TIF)

Figure S4 **Phylogenetic tree of Bcat family proteins.** Amino acid sequences of full-length proteins were analyzed. *ce*, *Caenorhabditis elegans*.
(TIF)

Figure S5 **Phylogenetic tree of Prdx family proteins.** Amino acid sequences of full-length proteins were analyzed.
(TIF)

Figure S6 **Phylogenetic tree of Frrs family proteins.** Amino acid sequences of full-length proteins were analyzed.
(TIF)

Figure S7 **Phylogenetic tree of Fth family proteins.** Amino acid sequences of full-length proteins were analyzed.
(TIF)

Figure S8 **Phylogenetic tree of Gclc family proteins.** Amino acid sequences of full-length proteins were analyzed.
(TIF)

Figure S9 **Phylogenetic tree of Gclm family proteins.** Amino acid sequences of full-length proteins were analyzed.
(TIF)

Figure S10 **Phylogenetic tree of Hmox family proteins.** Amino acid sequences of full-length proteins were analyzed.
(TIF)

Figure S11 **Expression of** ***frrs1c*** **and** ***hmox1c*** **in the liver.** Transverse sections of 5-dpf larvae through the trunk at the level of the liver (dotted line). Larvae were treated with (*frrs1c*, *hmox1a*) or without (*fabp10*) 100 µm DEM and analyzed by WISH before sectioning. Red and white arrowheads indicate positive and negative expression, respectively, of each gene in the liver. Asterisk denotes the basal expression in the intestine. Scale bar, 100 µm.
(TIF)

Figure S12 **Induction of Nrf2 target genes by sulforaphane.** 5-dpf larvae were treated with or without 40 µM sulforaphane for indicated hours and expression of seven Nrf2 target genes was analyzed by WISH. Lateral and ventral views. Red and white arrowheads indicate positive and negative expression, respectively, of each gene in the nose, gill and liver. Asterisks denote basal expression in the intestine.
(TIF)

Table S1 **Oligonucleotide primers used for RT-PCR analyses.**
(DOC)

Table S2 **Oligonucleotide primers used for plasmid construction.**
(DOC)

Table S3 **Identification of DEM-inducible genes in zebrafish (1).**
(DOC)

Table S4 **Identification of DEM-inducible genes in zebrafish (2).**
(DOC)
