# Correction: Tissue-Restricted Expression of Nrf2 and Its Target Genes in Zebrafish with Gene-Specific Variations in the Induction Profiles

**DOI:** 10.1371/annotation/50ee3aff-3010-4c42-a130-70509c88a67e

**Published:** 2012-09-14

**Authors:** Hitomi Nakajima, Yaeko Nakajima-Takagi, Tadayuki Tsujita, Shin-Ichi Akiyama, Takeshi Wakasa, Katsuki Mukaigasa, Hiroshi Kaneko, Yutaka Tamaru, Masayuki Yamamoto, Makoto Kobayashi

RT-PCR analyses was carried out 6 years ago when the identification of microarray genes were difficult due to poor zebrafish genome information. As such, some of the genes/proteins listed in the manuscript are no longer considered to be Nrf2 targets and the following corrections need to be made:

In paragraph three of the Results section, the sentence “Thirty-three genes were analyzed out of 46 selected genes, and 19 genes were identified to be induced by DEM" should read "Twenty-seven genes were analyzed out of 46 selected genes, and 17 genes were identified to be induced by DEM".

In Figure 1, the results of fr89g03.y1 and gtf3ab were deleted. The gene A2BFS9 should be renamed to si:ch211-117m20.5. The corrected Figure 1 can be viewed here: 

**Figure:**
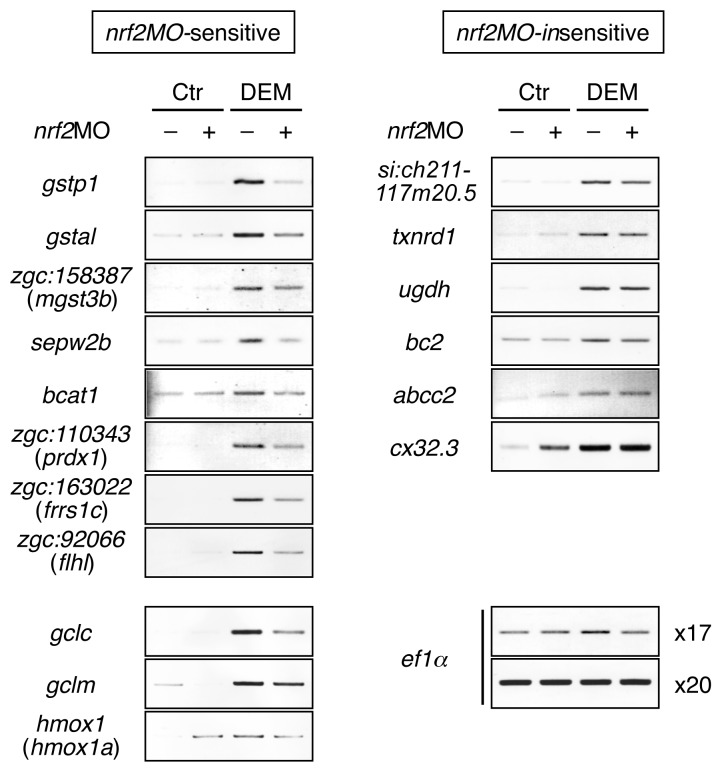


In Supplemental Table S1, the primer information of col5a1, rpap1, si:dkey-127j5.5, fr89g03.y1, gtf3ab, and zgc:162925 was deleted, and that of tubgcp3 was added. The genes zgc:92254 and zgc:136371 should be renamed to gsto1 and cyp2x10.2, respectively. The corrected Table S1 can be viewed here: 

In Supplemental Table S2, the reverse primer sequence and the cloning site of hmox1a were corrected. The corrected Table S2 can be viewed here: 

In Supplemental Table S3, the genes zgc:175127, si:ch211-140f21.1, zgc:101897, zgc:92254, and zgc:136371 should be renamed to wu:fc76h09, si:dkey-21e7.2, gsto2, gsto1, and cyp2x10.2, respectively. The unnamed 13th, 24th, and 42nd genes were newly named as zbtb34, si:ch211-191j22.3, and nuak2, respectively. The names of the 28th and 34th genes, si:rp71-4m17.1 and wu:fj04c06 respectively, were deleted. The product and/or chromosome information of wu:fc76h09, wu:fc01d01, si:dkey-21e7.2, zbtb34, gsto2, gsto1, zgc:162925, cyp2x10.2, and nuak2, were added or renewed. The RT-PCR results of col5a1, rpap1, si:dkey-127j5.5, si:ch211-191j22.3, gtf3ab, and zgc:162925 was deleted, and those of tubgcp3 were added. The corrected Figure S3 can be viewed here: 
