# Supplementary figures and images for: Tissue-Restricted Expression of Nrf2 and Its Target Genes in Zebrafish with Gene-Specific Variations in the Induction Profiles
Source: PLoS One. 2011 Oct 25;6(10):e26884. doi: 10.1371/journal.pone.0026884 (PMC3201981; doi:10.1371/journal.pone.0026884)

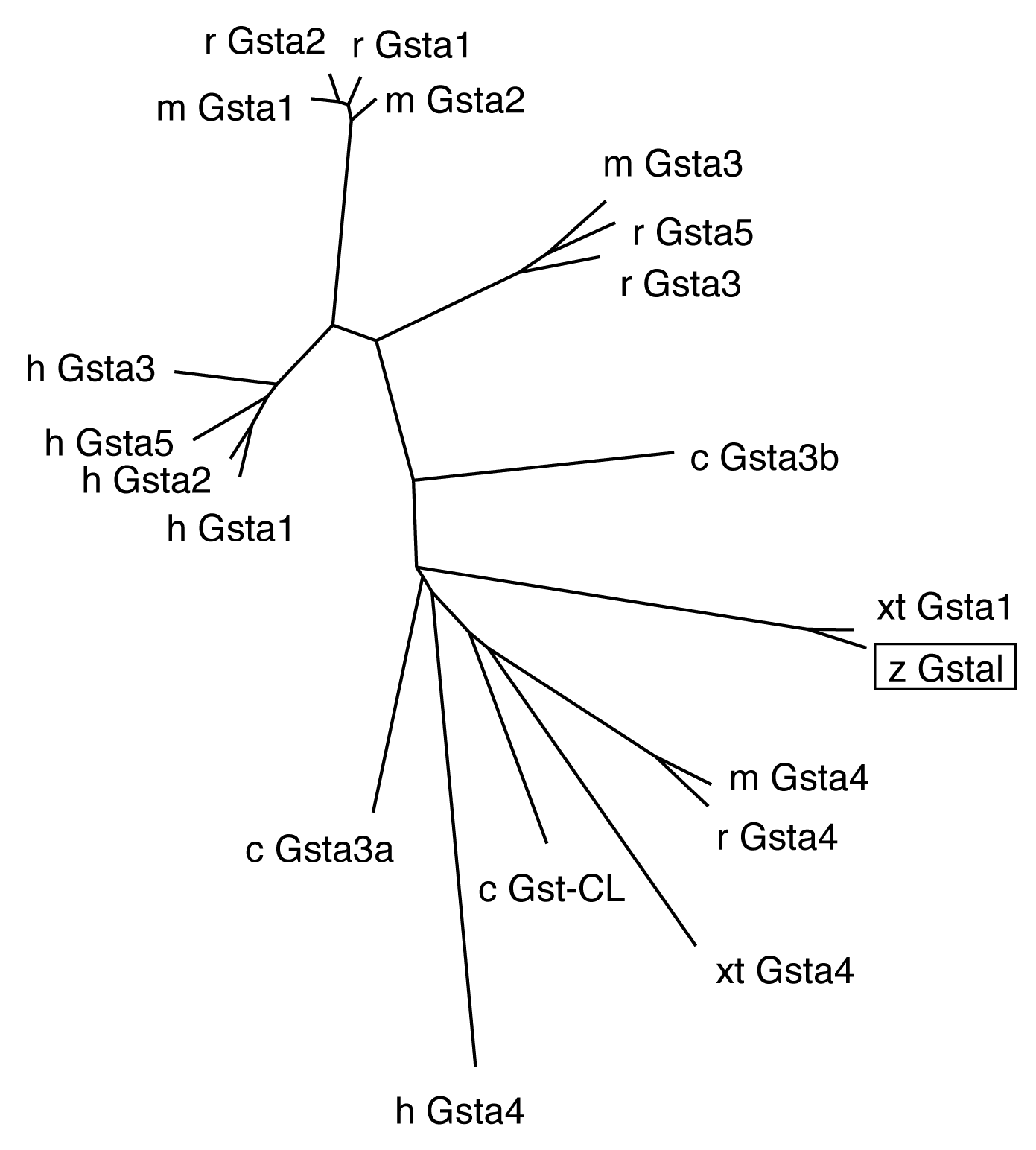

Supplement: Figure S1 — Phylogenetic tree of Gsta family proteins. Amino acid sequences of full-length proteins were analyzed. The tree was constructed by the neighbor-joining method using the ClustalW program (http://clustalw.ddbj.nig.ac.jp/top-j.html). c, chicken; h, human; m, mouse; r, rat; xt, Xenopus tropicalis; z, zebrafish. (TIF) [file pone.0026884.s001.tif]

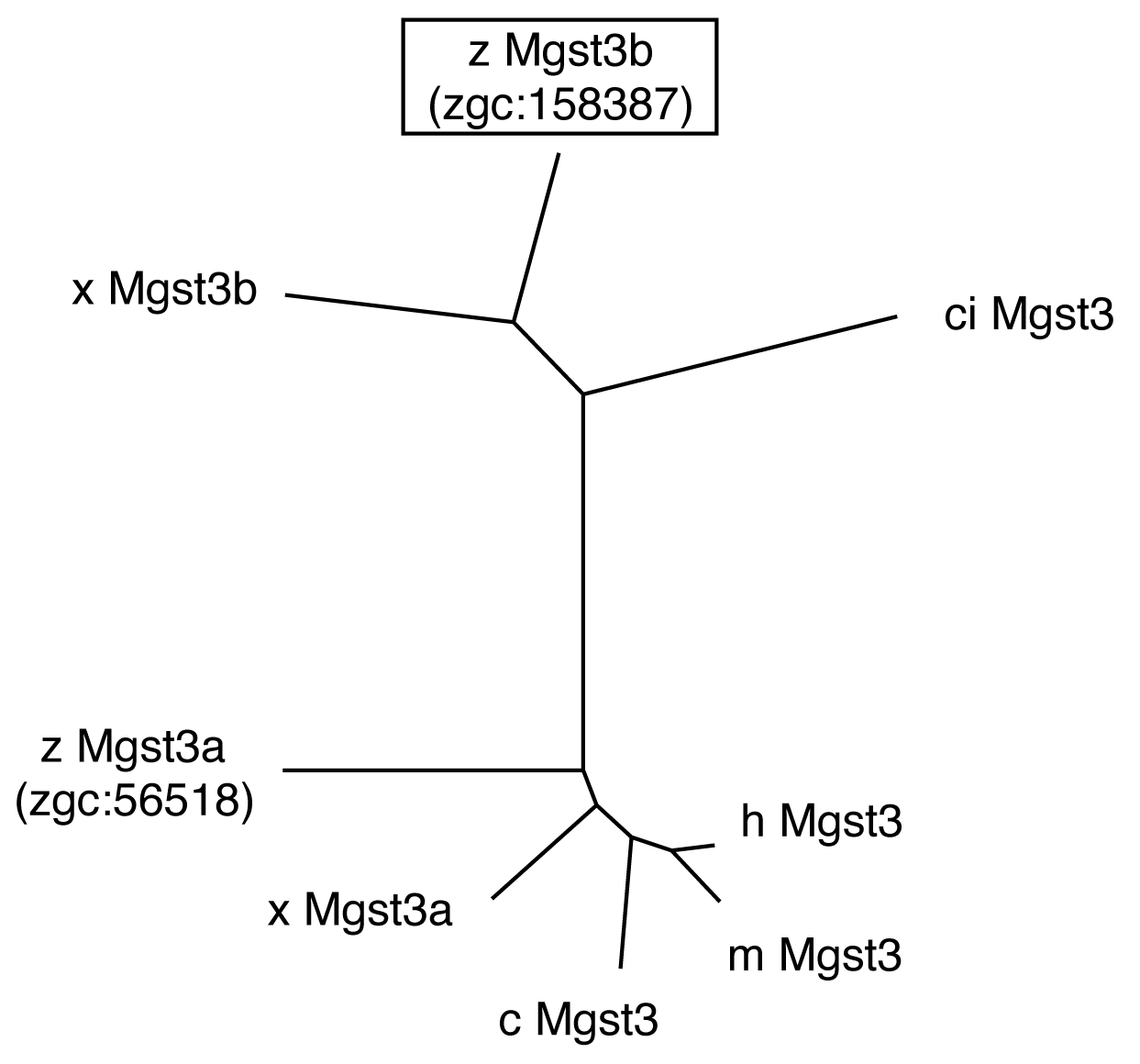

Supplement: Figure S2 — Phylogenetic tree of Mgst3 family proteins. Amino acid sequences of full-length proteins were analyzed. ci, Ciona intestinalis; x, Xenopus laevis. (TIF) [file pone.0026884.s002.tif]

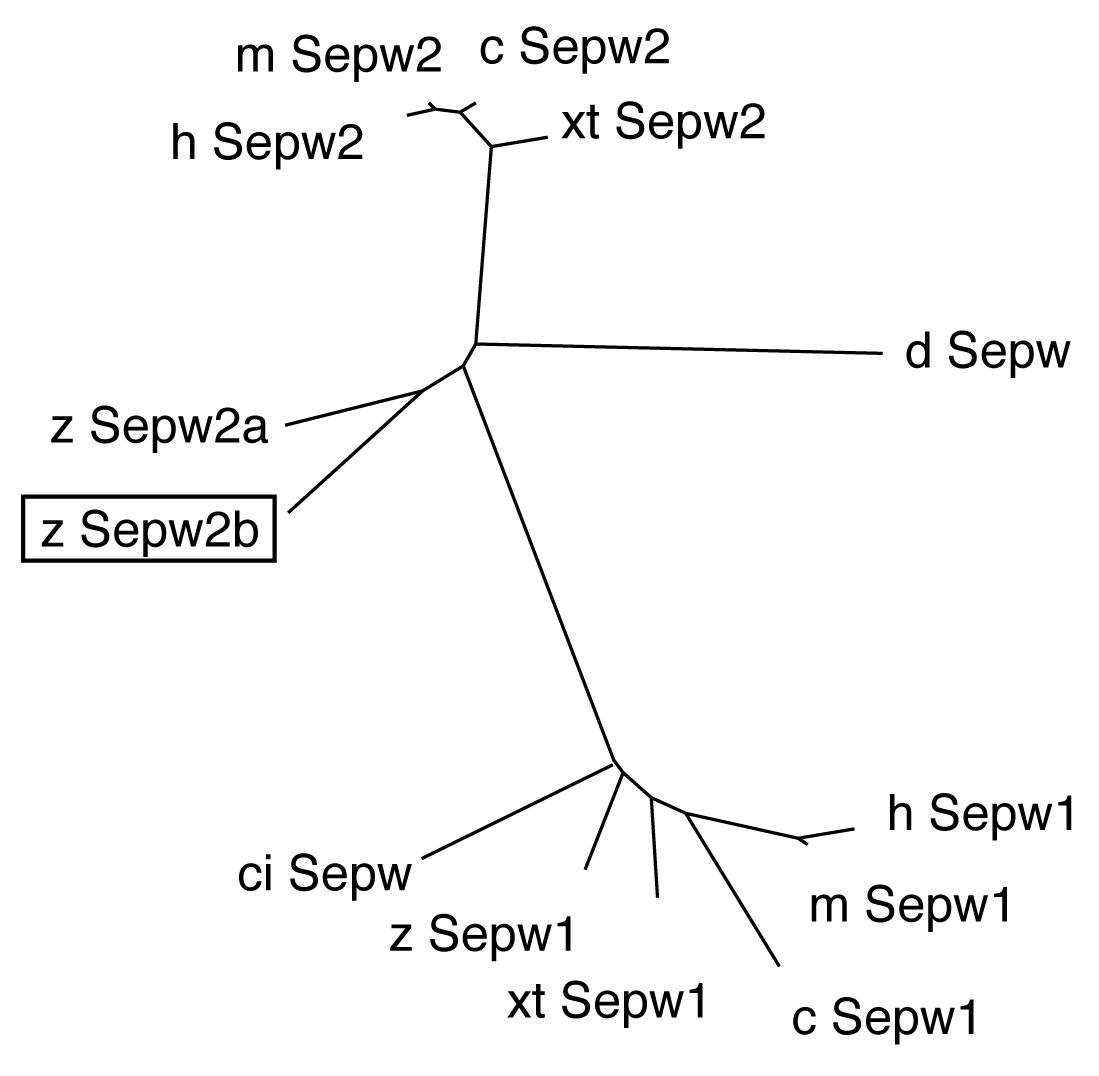

Supplement: Figure S3 — Phylogenetic tree of SepW family proteins. Amino acid sequences of full-length proteins were analyzed. d, Drosophila melanogaster. (TIF) [file pone.0026884.s003.tif]

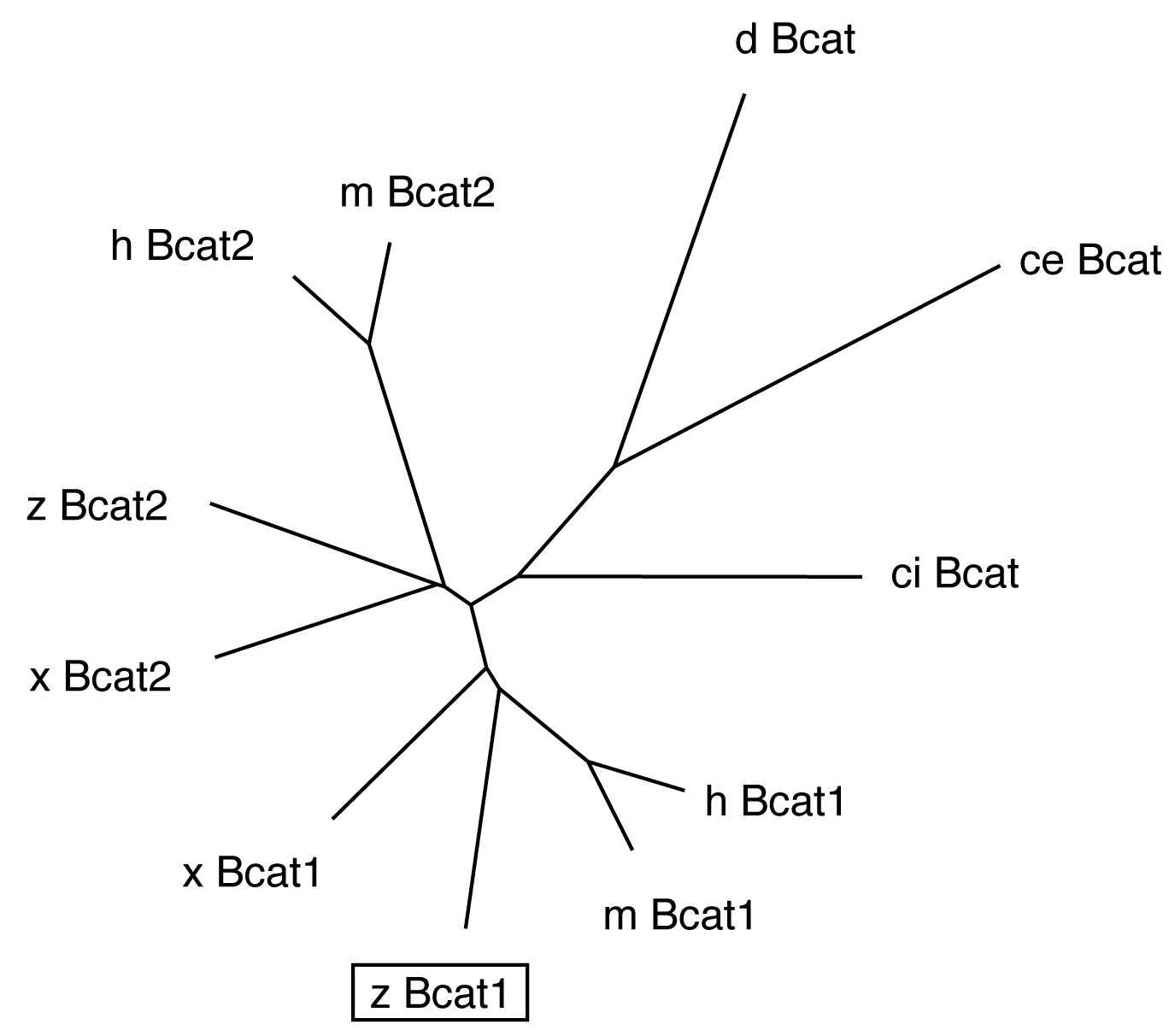

Supplement: Figure S4 — Phylogenetic tree of Bcat family proteins. Amino acid sequences of full-length proteins were analyzed. ce, Caenorhabditis elegans. (TIF) [file pone.0026884.s004.tif]

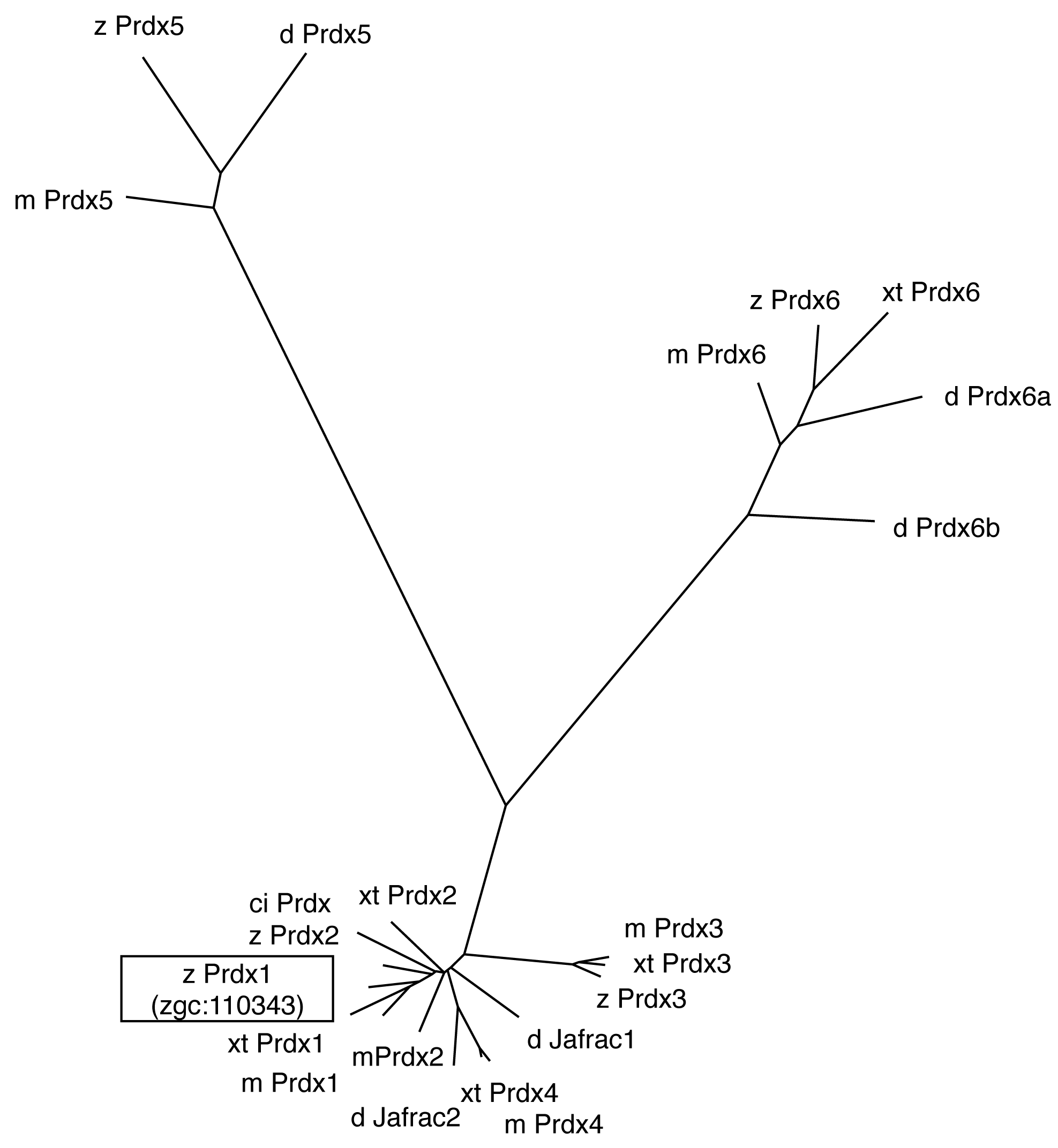

Supplement: Figure S5 — Phylogenetic tree of Prdx family proteins. Amino acid sequences of full-length proteins were analyzed. (TIF) [file pone.0026884.s005.tif]

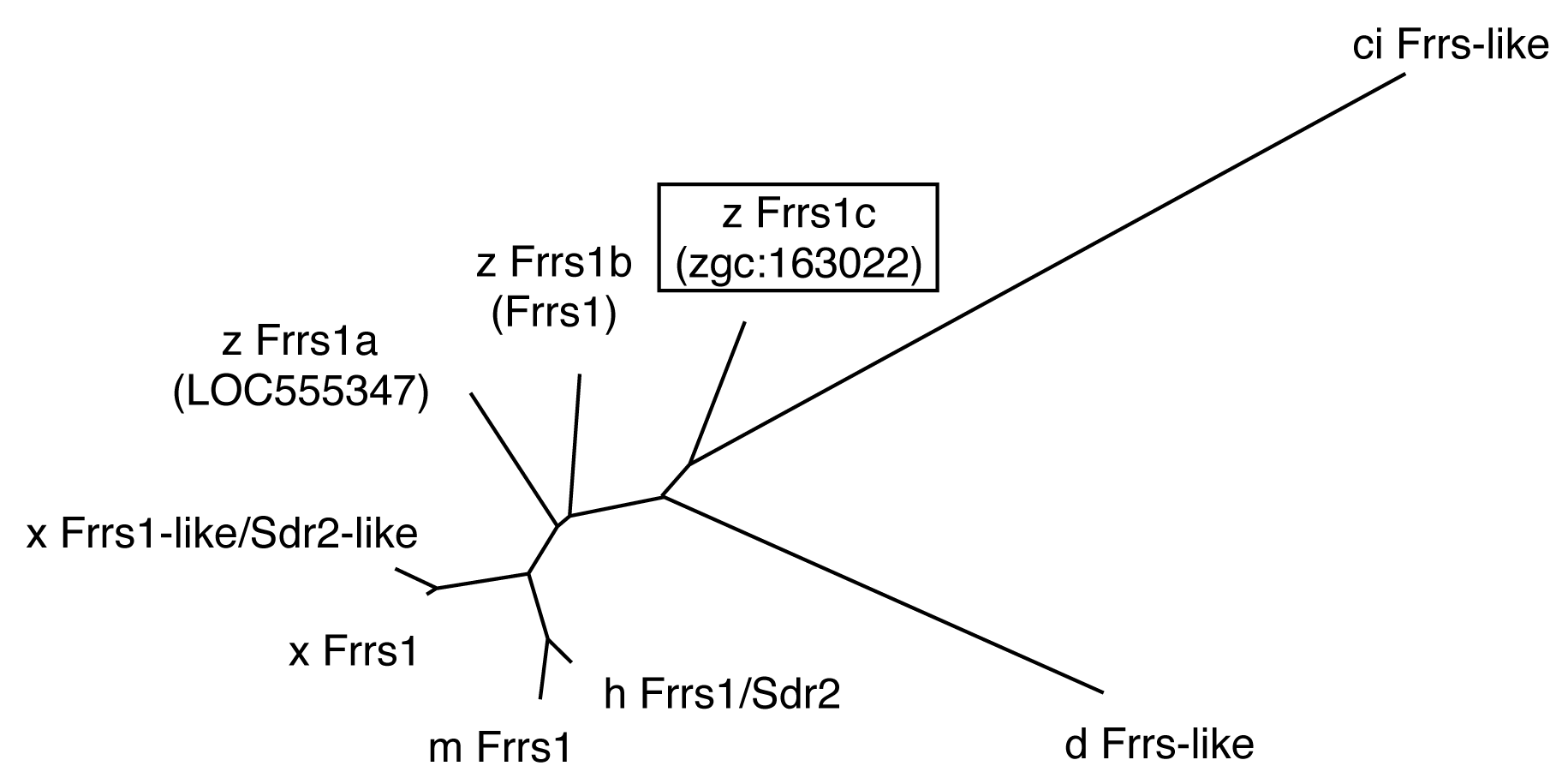

Supplement: Figure S6 — Phylogenetic tree of Frrs family proteins. Amino acid sequences of full-length proteins were analyzed. (TIF) [file pone.0026884.s006.tif]

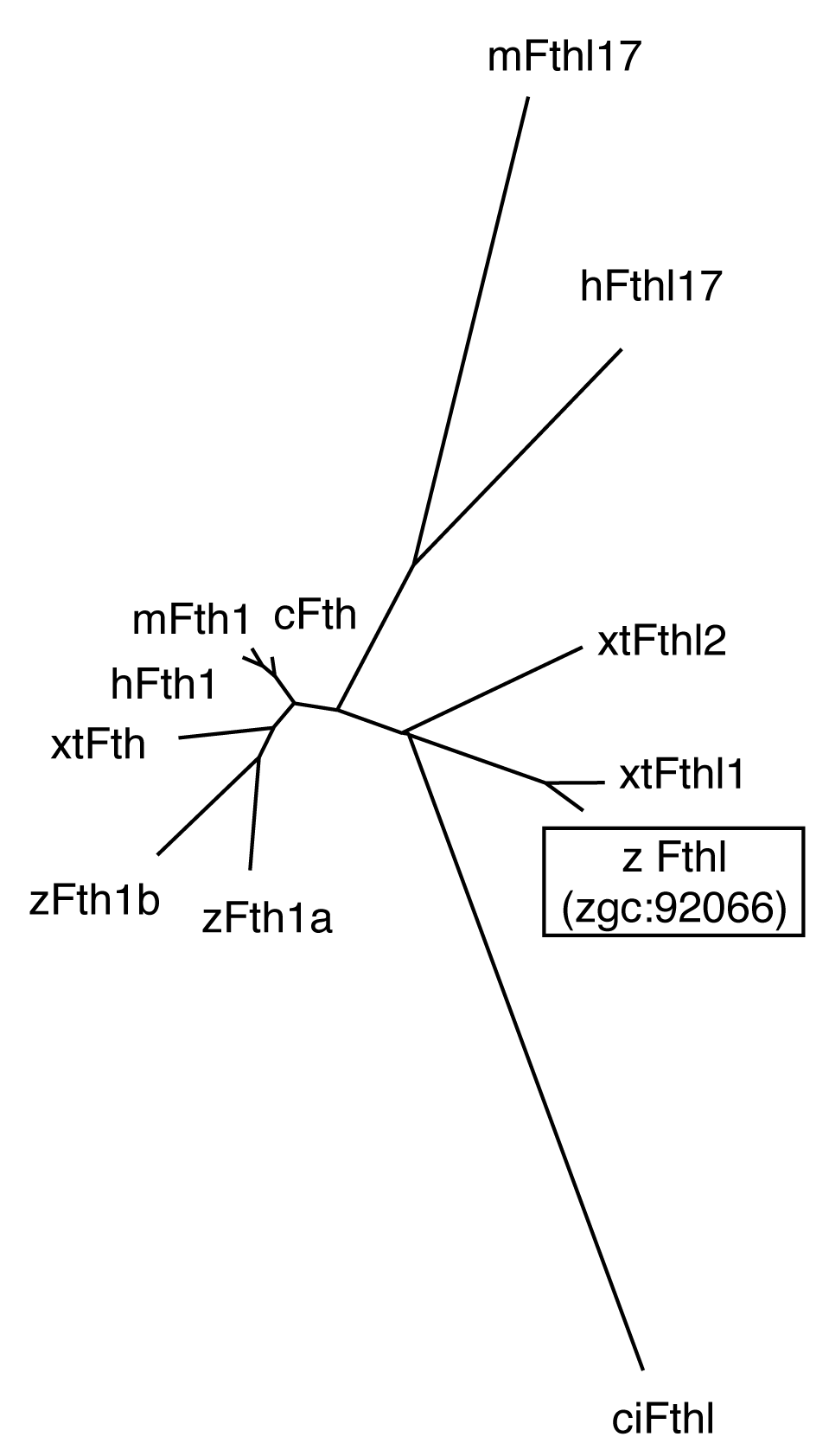

Supplement: Figure S7 — Phylogenetic tree of Fth family proteins. Amino acid sequences of full-length proteins were analyzed. (TIF) [file pone.0026884.s007.tif]

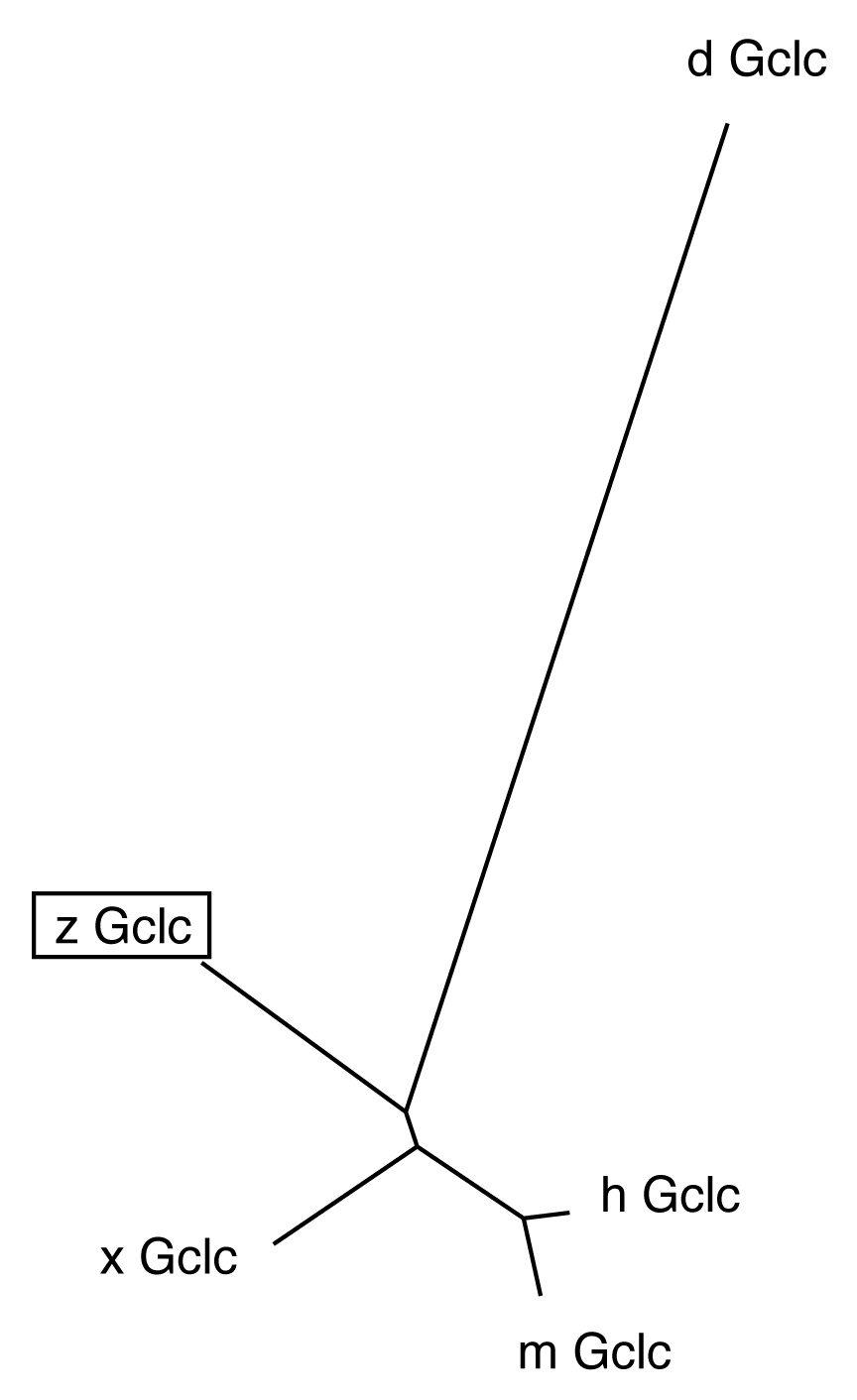

Supplement: Figure S8 — Phylogenetic tree of Gclc family proteins. Amino acid sequences of full-length proteins were analyzed. (TIF) [file pone.0026884.s008.tif]

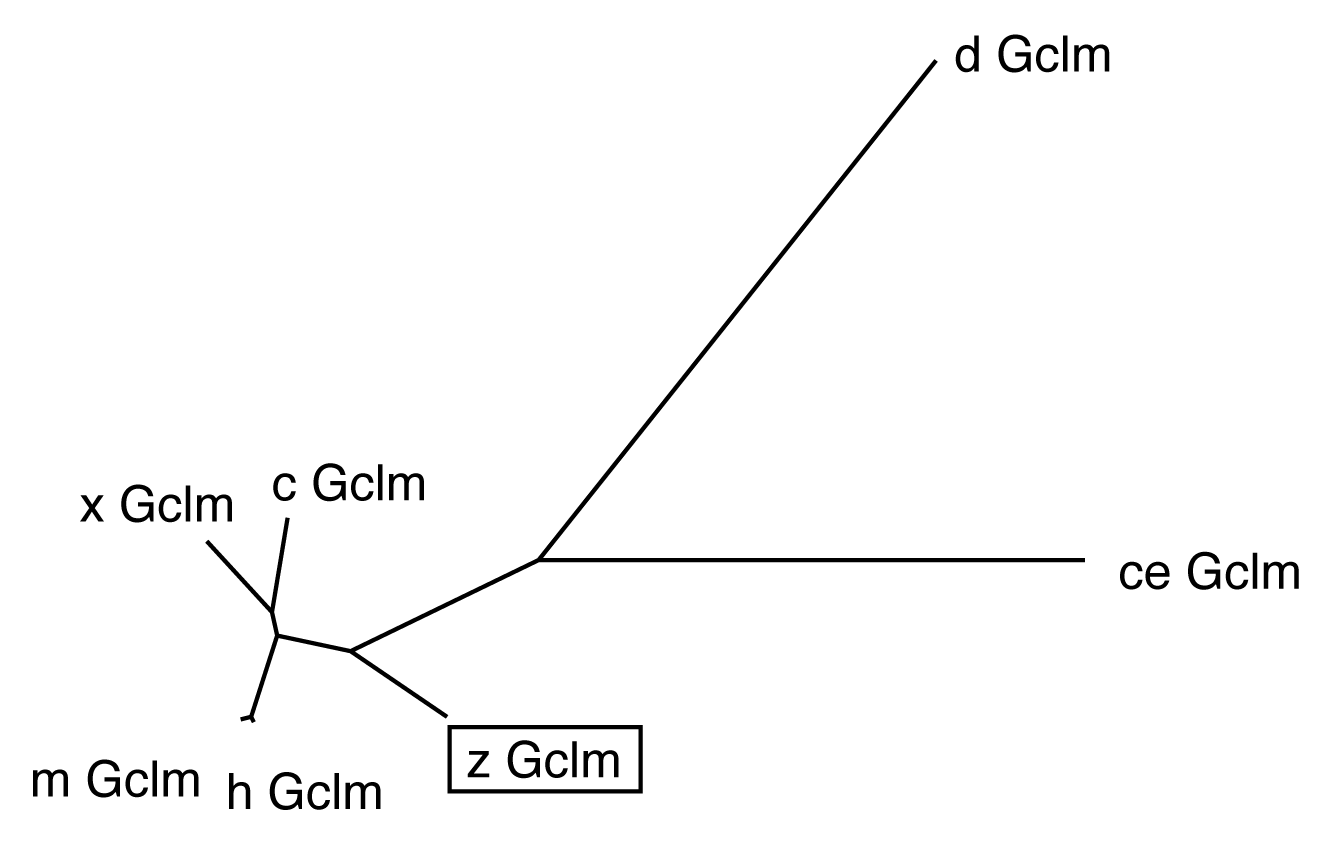

Supplement: Figure S9 — Phylogenetic tree of Gclm family proteins. Amino acid sequences of full-length proteins were analyzed. (TIF) [file pone.0026884.s009.tif]

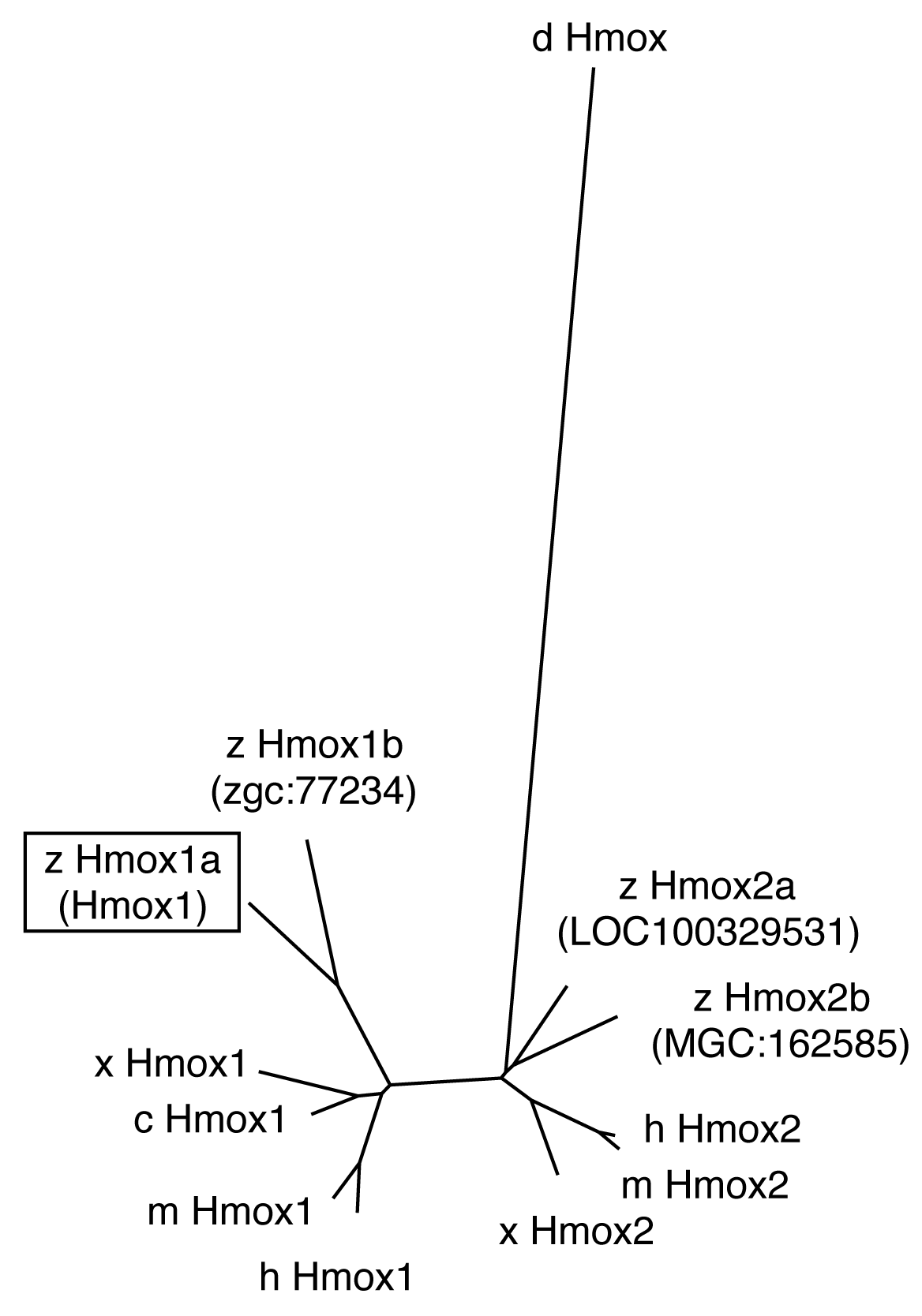

Supplement: Figure S10 — Phylogenetic tree of Hmox family proteins. Amino acid sequences of full-length proteins were analyzed. (TIF) [file pone.0026884.s010.tif]

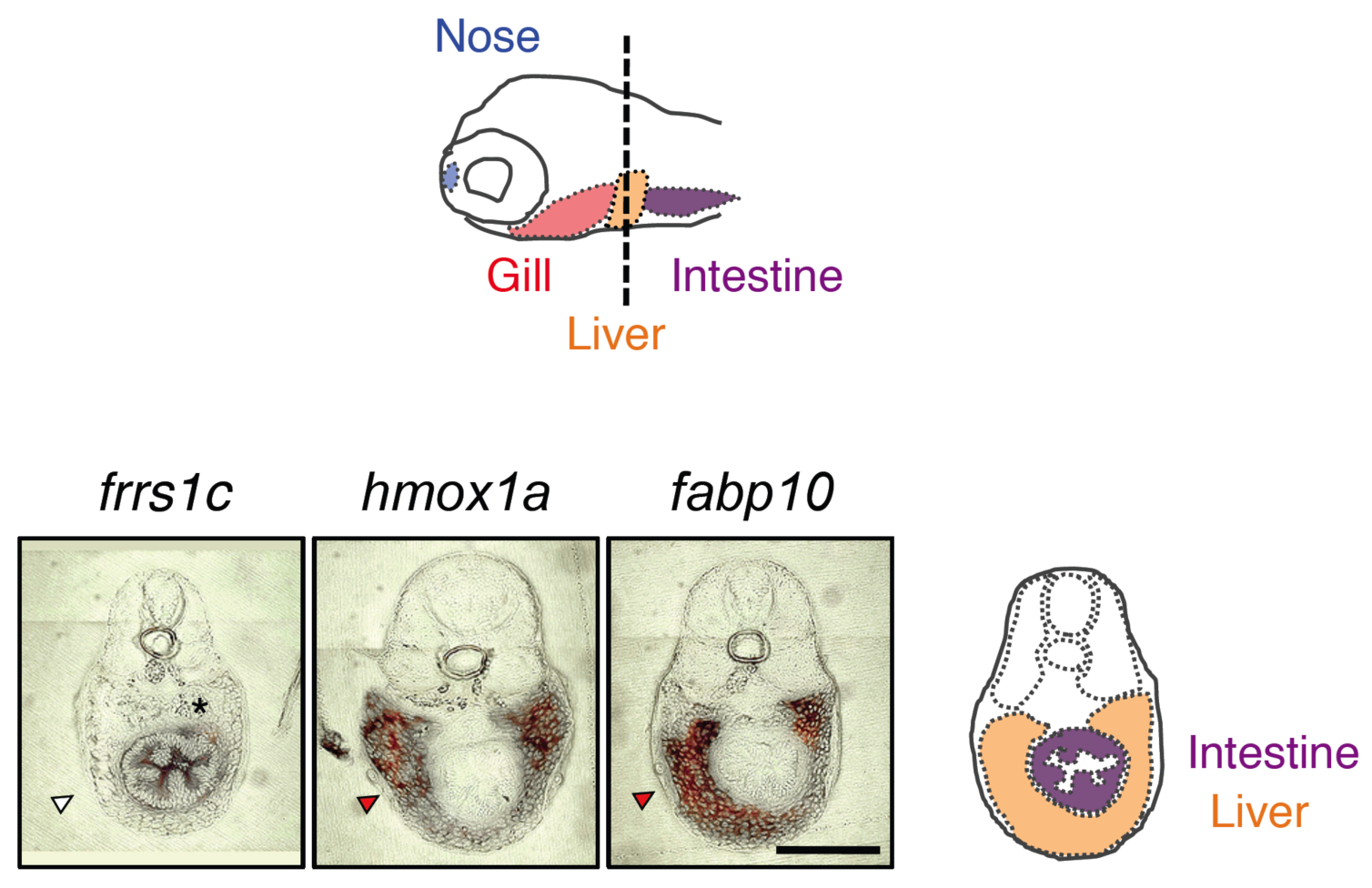

Supplement: Figure S11 — Expression of frrs1c and hmox1c in the liver. Transverse sections of 5-dpf larvae through the trunk at the level of the liver (dotted line). Larvae were treated with (frrs1c, hmox1a) or without (fabp10) 100 µm DEM and analyzed by WISH before sectioning. Red and white arrowheads indicate positive and negative expression, respectively, of each gene in the liver. Asterisk denotes the basal expression in the intestine. Scale bar, 100 µm. (TIF) [file pone.0026884.s011.tif]

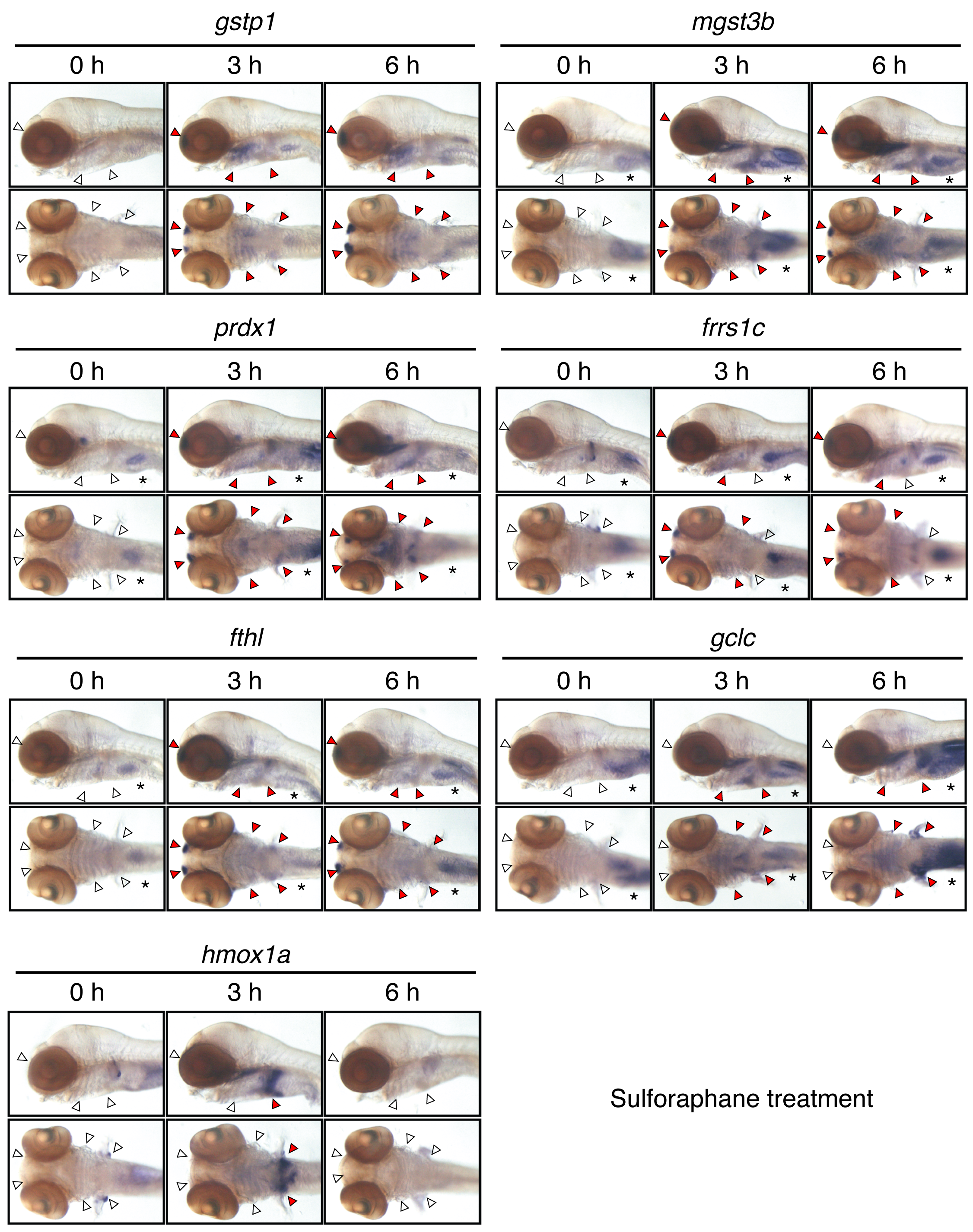

Supplement: Figure S12 — Induction of Nrf2 target genes by sulforaphane. 5-dpf larvae were treated with or without 40 µM sulforaphane for indicated hours and expression of seven Nrf2 target genes was analyzed by WISH. Lateral and ventral views. Red and white arrowheads indicate positive and negative expression, respectively, of each gene in the nose, gill and liver. Asterisks denote basal expression in the intestine. (TIF) [file pone.0026884.s012.tif]
