# Supplementary material for: Tissue-Restricted Expression of Nrf2 and Its Target Genes in Zebrafish with Gene-Specific Variations in the Induction Profiles
Source: PLoS One. 2011 Oct 25;6(10):e26884. doi: 10.1371/journal.pone.0026884 (PMC3201981; doi:10.1371/journal.pone.0026884)
Supplement: Table S1 — Oligonucleotide primers used for RT-PCR analyses. (DOC) [file pone.0026884.s013.doc]

**Table S1. Oligonucleotide primers used for RT-PCR analyses.**

| Gene | Forward primer | Reverse primer |
| --- | --- | --- |
| *gstp1* | 5’-CTAGGAGCAGCTTTGAAACGCACA | 5’-TGGCCAGAACATTTTCAAAGC |
| *gstal* | 5’-TCGATCCGGTGGCTCTTG | 5’-TTTGGGGAATGTGGCCAG |
| *atf4b2* | 5’-TGGGCTTGATCCGGTGTC | 5’-CGGGTCGGGGACAAAGAG |
| *mgst3b* (*zgc:158387*) | 5’-CCCAGCCAACTTCGGCTA | 5’-GACTCCGAACAGCCCGAT |
| *si:ch211-117m20.5* | 5’-TTGTTGCCTTTCCCAGGG | 5’-GCGAAACGAAATTCGGCA |
| *zgc:113006* | 5’-GCCGCTCGTCGAATAAAC | 5’-ACCATTGCCCACCGTAAC |
| *nme4* | 5’-GATTTGGTCTCCGCGTCC | 5’-CGAGATCTCCCTCTGCGC |
| *txnrd1* | 5’-CATGGCTGACCGTGCAGG | 5’-TGGGTGTCCACAGCTGCC |
| *sepw2b* | 5’-CATGAGGTTACGAGCCTCGCTT | 5’-ACGCTGATAAGGATCTCCGCTG |
| *bcat1* | 5’-CCAGGCTGTTCTGCCCTG | 5’-TGCCGTGTAACACCTGGC |
| *col5a1* | 5’-CATGACGTTTTCCTGGACACCA | 5’-TCAGATGTTGGAGCTCCGTCTG |
| *ugdh* | 5’-TCCGGAGGGTCAAAGGGC | 5’-TCCGGAGATGCCGGGTTG |
| *­rpap1* | 5’-GGTGCTGGAGCAGAAACTGGAT | 5’-TCGTAGTCAGGGTTGATGGGGT |
| *si:dkey-127j5.5* | 5’-CGGATACAGCGGCGGATA | 5’-AGGCCAACCGCATGGATC |
| fr89g03.y1 | 5’-GCGCTGACTGTGGCCTGC | 5’-CCGTGTTGGCATGCAGGG |
| *zgc:92254* | 5’-GCGCATGATGGAGGATCC | 5’-GCAATTGCCAAAACGTCG |
| *bc2* | 5’-AGAACGGCAGAGACTGGAGC | 5’-GCCAGCGTAGGCTGCG |
| *prdx1* (*zgc:110343*) | 5’-GCCCGCGAGTTCACTTTC | 5’-GCTTCCATCCGGCTGGAC |
| *mtnr1ba* | 5’-TCATCAGGAACCGGACGG | 5’-ACGTCAGCGACCCCAGCG |
| *gtf3ab* | 5’-GTGGCTTGGGCTTCCCAG | 5’-GGGCGTGGCAGGTTTTCT |
| *abcc2* | 5’-AATCTGAGCGGTGGGCAG | 5’-CGGATCTTTCTGAGCGCG |
| *cdkn1b* | 5’-CCGAGAGCCGAGGAAAAG | 5’-CGAGACGCTCCATGGAAG |
| *zgc:162925* | 5’-CGCTGTCCTGTCCAGCGG | 5’-TGCGGCCGTATTTCTTGG |
| *cebpg* | 5’-CCAAATGAGCAAGCAGTTGC | 5’-GAGCCTTCACATTGCAAACG |
| *frrs1c* (*zgc:163022*) | 5’-CCCAACCACCAGCAACGG | 5’-GCCCTTGGCGACTCCCTT |
| *fthl* (*zgc:92066*) | 5’-TACGACCGCGACTGCGAG | 5’-TGGCTGCAGATGATCCGA |
| *cx32.3* | 5’-ACCGACAGCCAAGGCCAC | 5’-GCCCCATCCAGGCTTCCT |
| *zgc:136371* | 5’-GGGGGCAAAGGGGTCATC | 5’-CTCTGGGCTCCCCAGCAA |
| *gclc* | 5’-CCAAGAAACATGCTGACCAC | 5’-GTCAGAGTGCTGAATCTTGG |
| *gclm* | 5’-GGGAATTCGAAGAAATGTCCGTCTTCGC | 5’-GGCTCGAGGTGTCAGCAACTGAATGTCG |
| *hmox1a* (*hmox1*) | 5’-GGTCGACTTAAAAAGCGTAAACTCCCATGC | 5’-GGAATTCATGGACTCCACCAAAAGCAAAG |
| *nqo1* | 5’-CCACTTCACACGCGAGGG | 5’-CCAGTGCAAACCCTCGCG |
| *ef1α* | 5’-GCCCCTGCCAATGTA | 5’-GGGCTTGCCAGGGAC |
