# Supplementary material for: Tissue-Restricted Expression of Nrf2 and Its Target Genes in Zebrafish with Gene-Specific Variations in the Induction Profiles
Source: PLoS One. 2011 Oct 25;6(10):e26884. doi: 10.1371/journal.pone.0026884 (PMC3201981; doi:10.1371/journal.pone.0026884)
Supplement: Table S2 — Oligonucleotide primers used for plasmid construction. (DOC) [file pone.0026884.s014.doc]

**Table S2. Oligonucleotide primers used for plasmid construction.**

| Gene | Forward primer | Reverse primer | Cloning sites |
| --- | --- | --- | --- |
| *gstal* | 5’-GGGAATTCGTCCGGGAAAGTCGTGCTG | 5’-GGCTCGAGACTTGAAGAGGTGGCTCAAC | *Eco*RI–*Xho*I |
| *mgst3b*  (*zgc:158387*) | 5’-GGGAATTCGGTCAGGTCCTGATCAGTTC | 5’-GGCTCGAGCATTAAAGGGATGGCTCACC | *Eco*RI– *Xho*I |
| *sepw2b* | 5’-GGGAATTCGTAACCAGCAACCTAAAGAC | 5’-GGGGATCCAATGAGGCTATAGGTGTGTAG | *Eco*RI–*Bam*HI |
| *bcat1* | 5’-GGGAATTCTTAGCACAAACCCATAAGCC | 5’-GGCTCGAGCCTGATATAAGATCCTGCCG | *Eco*RI– *Xho*I |
| *prdx1*  (*zgc:110343*) | 5’-GGGAATTCCTCGGATTGTGAAGACAGTTC | 5’-GGGGATCCTTGGAGAAGAAGTCTTTGCTC | *Eco*RI–*Bam*HI |
| *frrs1c*  (*zgc:163022*) | 5’-GGGAATTCGAATGGACTTGTAAGCGAGG | 5’-GGCTCGAGATCCAGCAGCGATCATCAGG | *Eco*RI– *Xho*I |
| *fthl*  (*zgc:92066*) | 5’-GGGAATTCCTTCAACAGTGATTGAACGG | 5’-GGCTCGAGAGGTGACCAACAAGTCAGTG | *Eco*RI– *Xho*I |
| *gclc* | 5’-GGGAATTCCACATAACGTGAAACCGG | 5’-GGCTCGAGCAGCTTCTCCATCATCCTCT | *Eco*RI– *Xho*I |
| *gclm* | 5’-GGGAATTCGAAGAAATGTCCGTCTTCGC | 5’-GGCTCGAGGTGTCAGCAACTGAATGTCG | *Eco*RI– *Xho*I |
| *hmox1a*  (*hmox1*) | 5’-GGAATTCATGGACTCCACCAAAAGCAAAG | 5’-GGCTCGAGACTTGAAGAGGTGGCTCAAC | *Eco*RI– *Xho*I |
