# Supplementary material for: Tissue-Restricted Expression of Nrf2 and Its Target Genes in Zebrafish with Gene-Specific Variations in the Induction Profiles
Source: PLoS One. 2011 Oct 25;6(10):e26884. doi: 10.1371/journal.pone.0026884 (PMC3201981; doi:10.1371/journal.pone.0026884)
Supplement: Table S3 — Identification of DEM-inducible genes in zebrafish (1). (DOC) [file pone.0026884.s015.doc]

**Table S3. Identification of DEM-inducible genes in zebrafish (1).**

| Ratio | Gene symbol in ZFIN | Gene product | Ch | RT-PCR | |
| --- | --- | --- | --- | --- | --- |
|  |  |  |  | Induction | Nrf2-  dependent |
| 4.86 | *gstp1* | Glutathione *S*-transferase pi | 14 | Yes | Yes |
| 4.67 | *gstal* | Glutathione *S*-transferase alpha | 13 | Yes | Yes |
| 3.76 | *zgc:175127* | novel | 14 | – | – |
| 3.52 | *atf4b2* | Activating transcription factor 4 | 3 | No | – |
| 3.44 | *zgc:158387* | Microsomal glutathione *S*-transferase 3 | 13 | Yes | Yes |
| 3.42 | *pc* | Pyruvate carboxylase | 7 | – | – |
| 3.13 | *si:ch211-117m20.5* | novel | 5 | Yes | No |
| 3.02 | *­wu:fc01d01* | EST (fc01d01.x1) | 13 | – | – |
| 2.99 | *zgc:113006* | Amine oxidase, copper containing 3 | 3 | No | – |
| 2.94 | *nme4* | Nucleoside diphosphate kinase | 3 | No | – |
| 2.89 | *si:ch211-140f21.1* | Complement component 4-like | 25 | – | – |
| 2.84 | *zgc:158614* | Apoptosis-inducing factor, mitochondrion-associated, 3 | 15 | – | – |
| 2.72 | *–* | EST (fp18f11.x1) | ND | – | – |
| 2.71 | *txnrd1* | Thioredoxin reductase 1 | 6 | Yes | No |
| 2.62 | *sepw2b* | Selenoprotein W 2b | 3 | Yes | Yes |
| 2.56 | *tubgcp3* | Tublin γ complex associated protein 3 | 1 | – | – |
| 2.44 | *bcat1* | Branched chain aminotransferase 1 | 4 | Yes | Yes |
| 2.42 | *col5a1* | Collagen, type V, α1 | 21 | No |  |
| 2.41 | *ugdh* | UDP-glucose dehydrogenase | 1 | Yes | No |
| 2.37 | *zgc:101897* | Glutathione S-transferase omega | 13 | – | – |
| 2.37 | *wu:fb11a02* | EST (fb11a02.x1) | 13 | – | – |
| 2.36 | *­rpap1* | RNA polymerase II associated protein 1 | 17 | No | – |
| 2.35 | *si:dkey-127j5.5* | Activating transcription factor 5 | 5 | No | – |
| 2.35 | *–* | novel (fr89g03.y1) | 21 | Yes | No |
| 2.34 | *zgc:92254* | Glutathione S-transferase omega | 13 | No | – |
| 2.34 | *pard3* | Par-3 partitioning defective 3 homolog | 2 | – | – |
| 2.31 | *bc2* | Chromatin modifying protein 2A | 12 | Yes | No |
| 2.30 | *si:rp71-4m17.1* | Neuromedin U | 20 | – | – |
| 2.25 | *zgc:110343* | Peroxiredoxin 1 | 2 | Yes | Yes |
| 2.23 | *mtnr1ba* | Melatonin receptor type 1B | 15 | No | – |
| 2.23 | *gtf3ab* | General transcription factor IIIA | 24 | Yes | No |
| 2.23 | *abcc2* | ATP-binding cassette C2 | 13 | Yes | No |
| 2.22 | *cdkn1b* | Cyclin-dependent kinase inhibitor 1 | 25 | No | – |
| 2.08 | *wu:fj04c06* | novel (fj04e06.x1) | 1 | – | – |
| 2.08 | *zgc:162925* | Solute carrier family 6, member 15 | 24 | No | – |
| 2.07 | *fah* | Fumarylacetoacetate hydrolase | 7 | – | – |
| 2.07 | *cebpg* | CCAAT/enhancer binding protein gamma | 7 | No | – |
| 2.06 | *zgc:163022* | Ferric-chelate reductase 1 | 24 | Yes | Yes |
| 2.06 | *zgc:92066* | Ferritin heavy chain | 3 | Yes | Yes |
| 2.06 | *cx32.3* | Connexin 32.3 | 20 | Yes | No |
| 2.02 | *zgc:136371* | Cytochrome P450 2F2 | 7 | No | – |
| 2.00 | – | EST (fp03d07.y1) | ND | – | – |
