# Supplementary material for: Tissue-Restricted Expression of Nrf2 and Its Target Genes in Zebrafish with Gene-Specific Variations in the Induction Profiles
Source: PLoS One. 2011 Oct 25;6(10):e26884. doi: 10.1371/journal.pone.0026884 (PMC3201981; doi:10.1371/journal.pone.0026884)
Supplement: Table S4 — Identification of DEM-inducible genes in zebrafish (2). (DOC) [file pone.0026884.s016.doc]

**Table S4. Identification of DEM-inducible genes in zebrafish (2).**

| Gene symbol in ZFIN | Gene product | Ch | RT-PCR | |
| --- | --- | --- | --- | --- |
|  |  |  | Induction | Nrf2-  dependent |
| *gclc* | Glutamate-cysteine ligase, catalytic subunit | 13 | Yes | Yes |
| *gclm* | Glutamate-cysteine ligase, modifier subunit | 8 | Yes | Yes |
| *hmox1* | Heme oxygenase (decycling) 1 | 3 | Yes | Yes |
| *nqo1* | NAD(P)H dehydrogenase, quinone 1 | 7 | No | – |
