# Supplementary figures and images for: Correction: Tissue-Restricted Expression of Nrf2 and Its Target Genes in Zebrafish with Gene-Specific Variations in the Induction Profiles
Source: PLoS One. 2012 Sep 14;7(9):10.1371/annotation/50ee3aff-3010-4c42-a130-70509c88a67e. doi: 10.1371/annotation/50ee3aff-3010-4c42-a130-70509c88a67e (PMC3472963; doi:10.1371/annotation/50ee3aff-3010-4c42-a130-70509c88a67e)

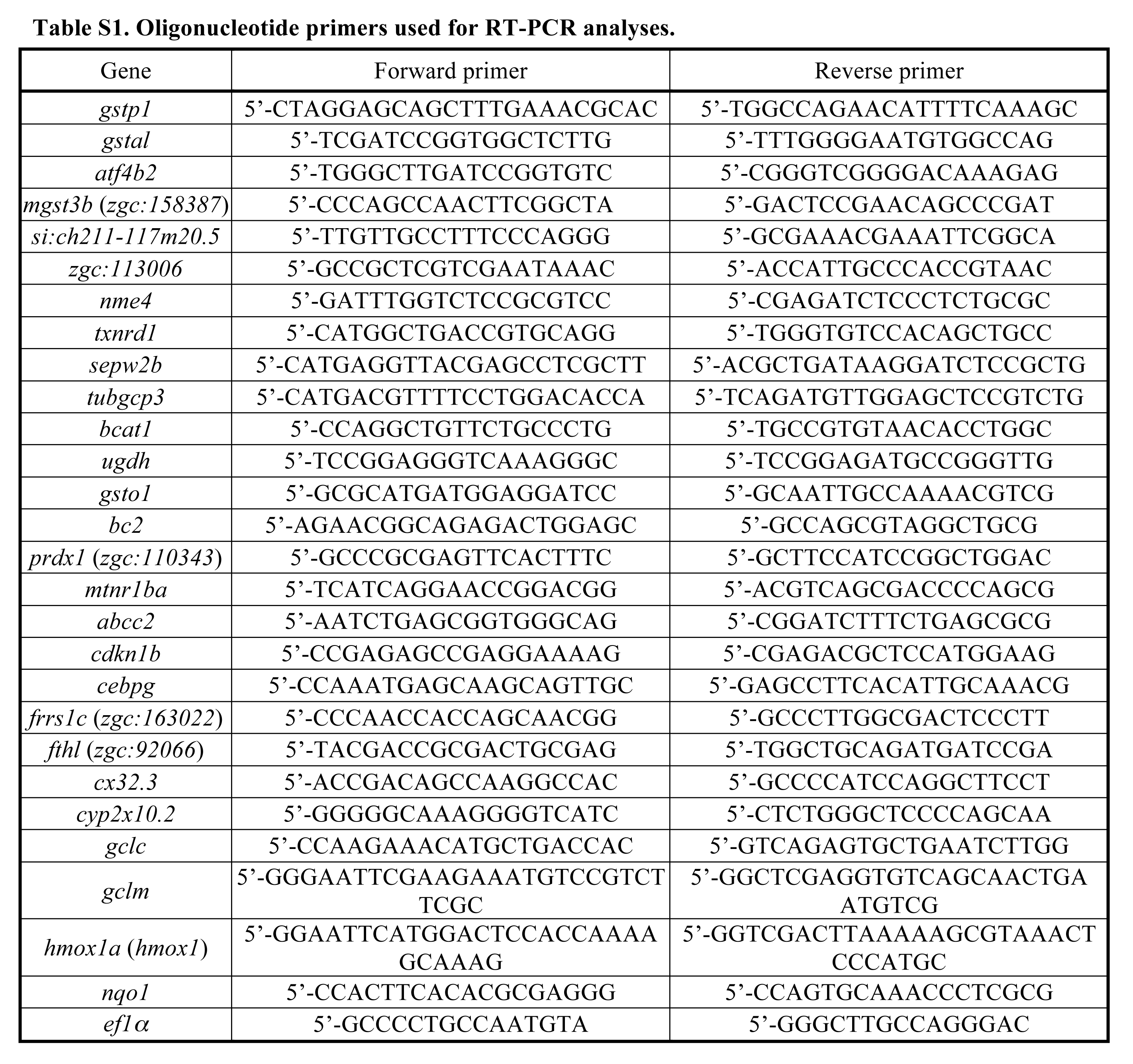

Supplement: Supplementary file 1 [file pone.50ee3aff-3010-4c42-a130-70509c88a67e.s001.tif]

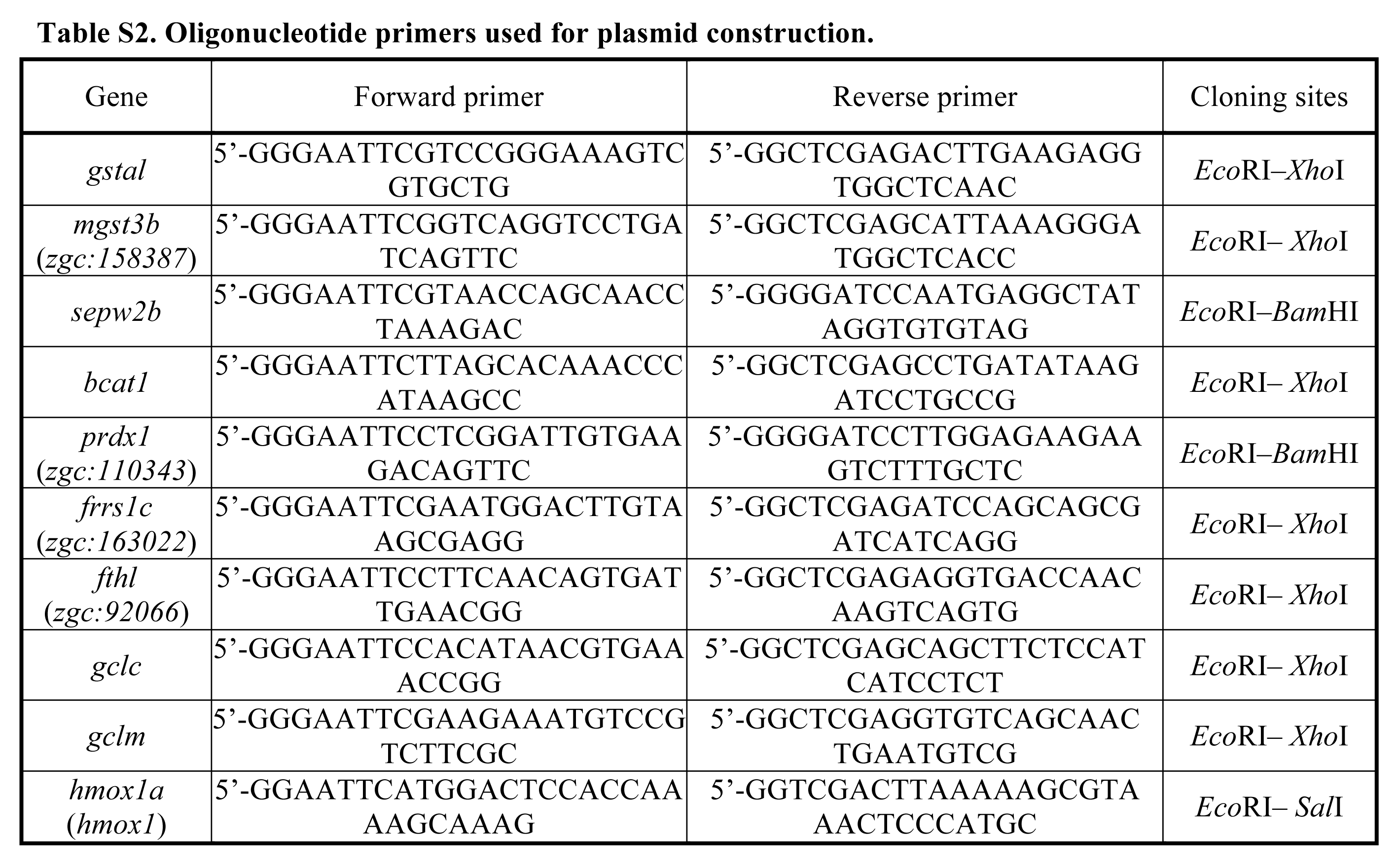

Supplement: Supplementary file 2 [file pone.50ee3aff-3010-4c42-a130-70509c88a67e.s002.tif]

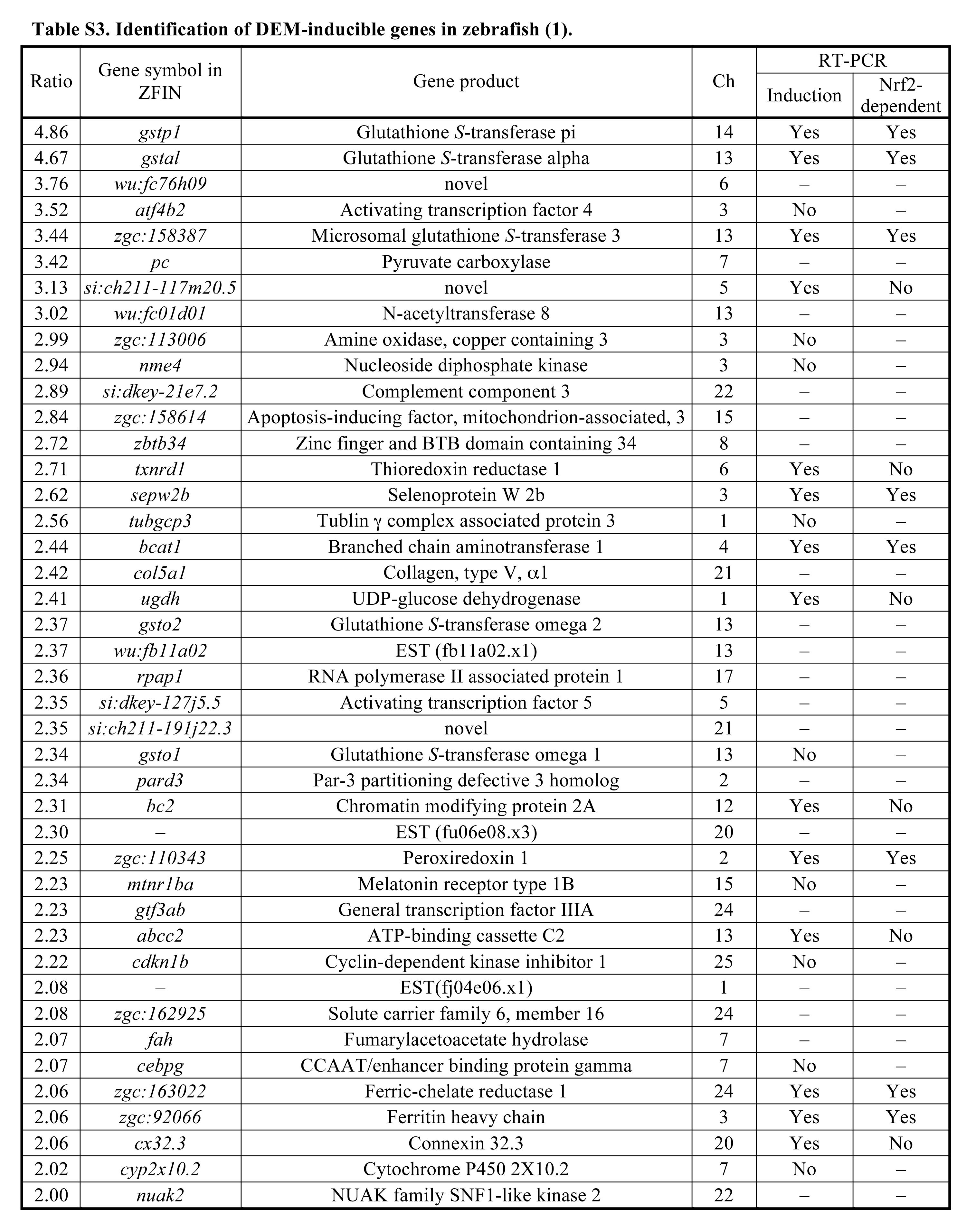

Supplement: Supplementary file 3 [file pone.50ee3aff-3010-4c42-a130-70509c88a67e.s003.tif]
